# Thermally activated delayed fluorescence emitters showing wide-range near-infrared piezochromism and their use in deep-red OLEDs†

**DOI:** 10.1039/d3sc05188a

**Published:** 2023-11-29

**Authors:** Pagidi Sudhakar, Abhishek Kumar Gupta, David B. Cordes, Eli Zysman-Colman

**Affiliations:** a Organic Semiconductor Centre, EaStCHEM School of Chemistry, University of St Andrews St Andrews UK KY16 9ST eli.zysman-colman@st-andrews.ac.uk

## Abstract

Organic small molecules exhibiting both thermally activated delayed fluorescence (TADF) and wide-ranging piezochromism (Δ*λ* > 150 nm) in the near-infrared region have rarely been reported in the literature. We present three emitters MeTPA-BQ, tBuTPA-BQ and TPPA-BQ based on a hybrid acceptor, benzo[g]quinoxaline-5,10-dione, that emit *via* TADF, having photoluminescence quantum yields, *Φ*_PL_, of 39–42% at photoluminescence (PL) maxima, *λ*_PL_, of 625–670 nm in 2 wt% doped films in 4,4′-bis(*N*-carbazolyl)-1,1′-biphenyl (CBP). Despite their similar chemical structures, the PL properties in the crystalline states of MeTPA-BQ (*λ*_em_ = 735 nm, *Φ*_PL_ = 2%) and tBuTPA-BQ (*λ*_em_ = 657 nm, *Φ*_PL_ = 11%) are significantly different. Further, compounds tBuTPA-BQ and TPPA-BQ showed a significant PL shift of ∼98 and ∼165 nm upon grinding of the crystalline samples, respectively. Deep-red organic light-emitting diodes with MeTPA-BQ and tBuTPA-BQ were also fabricated, which showed maximum external quantum efficiencies, EQE_max_, of 10.1% (*λ*_EL_ = 650 nm) and 8.5% (*λ*_EL_ = 670 nm), respectively.

## Abstract 2

We report rare examples of organic small molecules exhibiting both thermally activated delayed fluorescence (TADF) and wide-ranging piezochromism (Δ*λ* > 150 nm) in the near-infrared region and their utilization in deep-red OLEDs.

## Graphical abstract

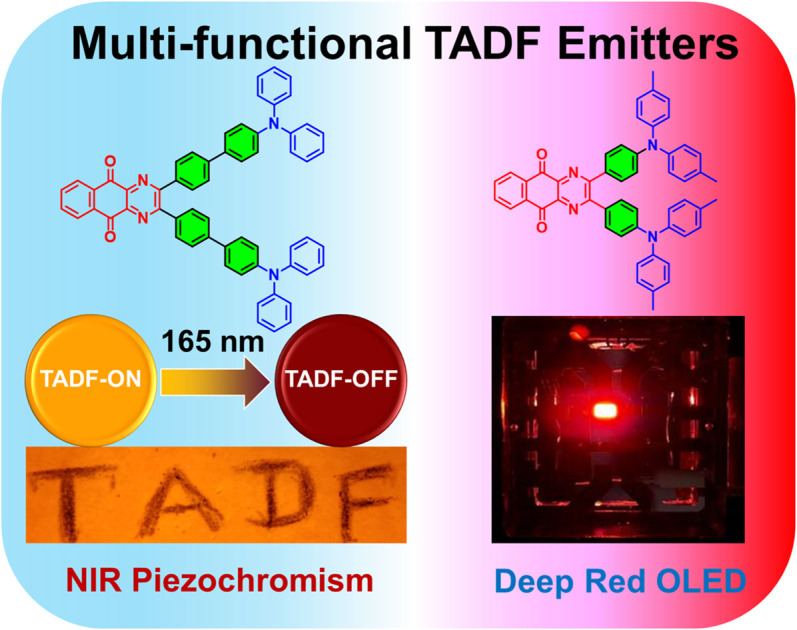

## Introduction

Piezochromism refers to a phenomenon in which a material exhibits a color change or emission shift in response to a mechanical stimulus, such as grinding, shearing, rubbing, or stretching,^1–3^ and has found diverse applications such as in security papers,^4^ sensors,^5^ memory devices,^6^ optical storage,^7^ and anti-counterfeiting.^8^ Typically, piezochromism is found to result from mechanical force-induced changes in the packing/intermolecular interactions of molecules in the solid state. Piezochromism or piezochromic luminescence (PCL) has been extensively investigated in numerous organic semiconductor materials.^2^ Of these, a subset emit *via* thermally activated delayed fluorescence (TADF).^9^ TADF compounds typically possess highly twisted donor–acceptor conformations, which ensures that there is a sufficiently small singlet–triplet energy gap, Δ*E*_ST_, to enable reverse intersystem crossing at ambient temperatures.^10^ Piezochromic TADF compounds also engage in weak non-covalent intermolecular interactions and thus their optical properties are most likely to respond to external mechanical stimulus. For instance, Xie *et al.*^11^ reported CPzPO and SPzPO that showed a dual emission in the crystalline state (*λ*_PL_ of 459 nm and 564 nm for CPzPO and 433 nm and 546 nm for SpzPO). After grinding, the lower energy emission band was enhanced while the higher energy emission band disappeared for both compounds, which is due to a crystalline to amorphous material transition. The lower energy band had TADF behavior, with *τ*_d_ of 62 and 29 μs, for CPzPO and SPzPO, respectively. Zhou *et al.*^12^ reported a tetracoordinate boron-based TADF emitter *R*-DOBP (*Φ*_PL_ of 11% and *τ*_d_ of 6 μs in the neat film). This compound showed a red-shifted emission from 580 nm to 647 nm upon grinding, caused by a crystalline to amorphous material transition. Swager and co-workers^13^ reported a through space charge transfer TADF emitter XPT that showed a change in *λ*_PL_ from 536 nm to 569 nm upon grinding, the original emission could be restored upon DCM solvent fuming. XPT emits at *λ*_PL_ of 566 nm, has a *Φ*_PL_ of 66% and a *τ*_d_ of 3.3 μs in 10 wt% doped films in DPEPO. An organic light-emitting diode (OLED) with XPT showed an EQE_max_ of 10% at *λ*_EL_ of 584 nm. Okazaki *et al.*^14^ reported multifunctional emitters 1 and 2 that showed both TADF and multi-colour mechanochromic luminescence. Upon grinding crystals of either 1_Y (yellow crystals of 1, *λ*_PL_ at 568 nm) or 1_O (orange crystals of 1, *λ*_PL_ at 640 nm), resulted in the formation of an amorphous form 1_R emitting a *λ*_PL_ of 673 nm. Sample 1_R was subjected to thermal annealing and DCM vapor produced 1_O2 (*λ*_PL_ of 646 nm) and 1_YO (*λ*_PL_ of 596 nm), respectively. The grinding of either of 1_O2 or 1_YO restored 1_R. Compound 2 also exhibited similar tricolor mechanochromic luminescence. The OLED with 10 wt% compound 1 doped in CBP showed an EQE_max_ of 16.8% at *λ*_EL_ of 613 nm.

**Fig. 1:**
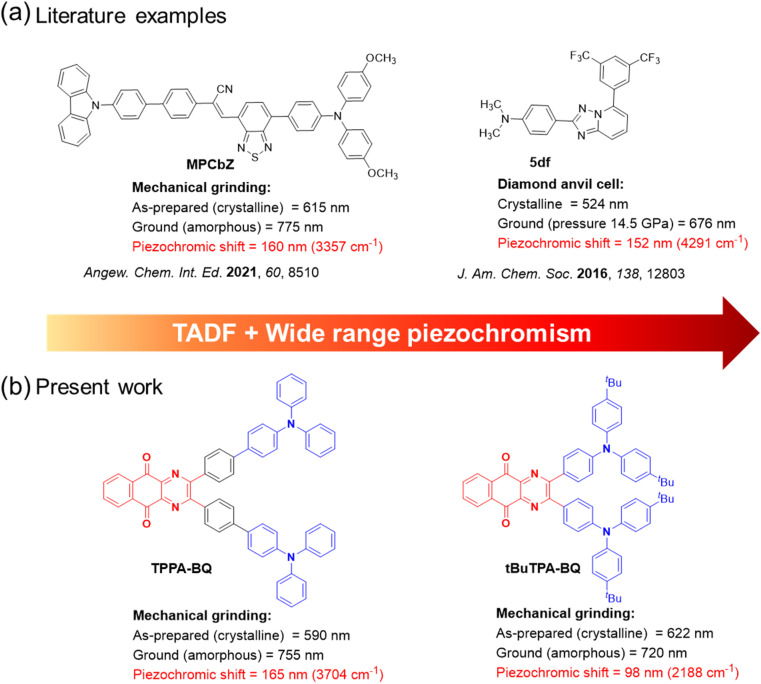
(a) Chemical structures of reported conventional wide range piezochromic materials. (b) Molecules exhibiting TADF and near-infrared piezochromism reported in the present study.

To date, the reported TADF-PCL materials have all showed a somewhat limited magnitude of PL spectral shift (<110 nm) upon grinding (Fig. S1†) and there are no reports of TADF-PCL compounds exhibiting near-infrared piezochromism. In fact, to our surprise, there are only a very limited number of metal-free near-infrared piezochromic organic materials with spectral shifts greater than 150 nm reported in the literature. For instance, Zhang and co-workers reported a near-infrared piezochromic material, MPCbZ, showing a large emission shift of 160 nm.^15^ The pristine solid (crystalline) emits at *λ*_PL_ of 615 nm (*τ*_PL_ = 4.4 ns) and has a *Φ*_PL_ of 60.7% (Fig. 1). Grinding led to a change in morphology of the solid to an amorphous powder, emitting at *λ*_PL_ of 775 nm (*τ*_PL_ = 2.6 ns) and having a much decreased *Φ*_PL_ of 5.2%. The original photophysics could be restored upon DCM solvent fuming. Wu *et al.* developed a library of mechanochromic materials in which the emission of a powder sample of compound 5df switched from a *λ*_PL_ of 539 to 588 nm when ground and where the original emission was restored upon fuming with different solvent vapors such as those from DCM, toluene, and acetone.^16^ However, when a pressure of 14.5 GPa was applied on the crystal using a diamond anvil cell, the emission change was more dramatic, shifting from 524 to 676 nm (Fig. 1).

In this work, we report three donor–acceptor TADF emitters consisting of a strong electron acceptor, benzo[*g*]quinoxaline-5,10-dione, composed of electron-withdrawing diketone and pyrazine moieties, linked to triarylamine donors, MeTPA-BQ, tBuTPA-BQ and TPPA-BQ (Fig. 1b). Compounds tBuTPA-BQ and TPPA-BQ were found to exhibit near-infrared piezochromism associated with large spectral shifts of ∼98 nm and ∼165 nm, respectively. The ground solids are stable under ambient conditions and can be heated to 200 °C without change in emission. The properties of the ground sample of TPPA-BQ could be restored to their original state upon ethyl acetate (EtOAc) fuming. As 2 wt% doped films in CBP, MeTPA-BQ, tBuTPA-BQ and TPPA-BQ all emit in the red and exhibit TADF, with *Φ*_PL_ of 42% at 650 nm, 41% at 670 nm, and 39% at 625 nm, respectively. MeTPA-BQ and tBuTPA-BQ were used as emitters in OLEDs, which showed maximum external quantum efficiencies (EQE_max_) of 10.1% at 650 nm and 8.5% at 670 nm, respectively.

## Results and discussions

**Scheme 1:**
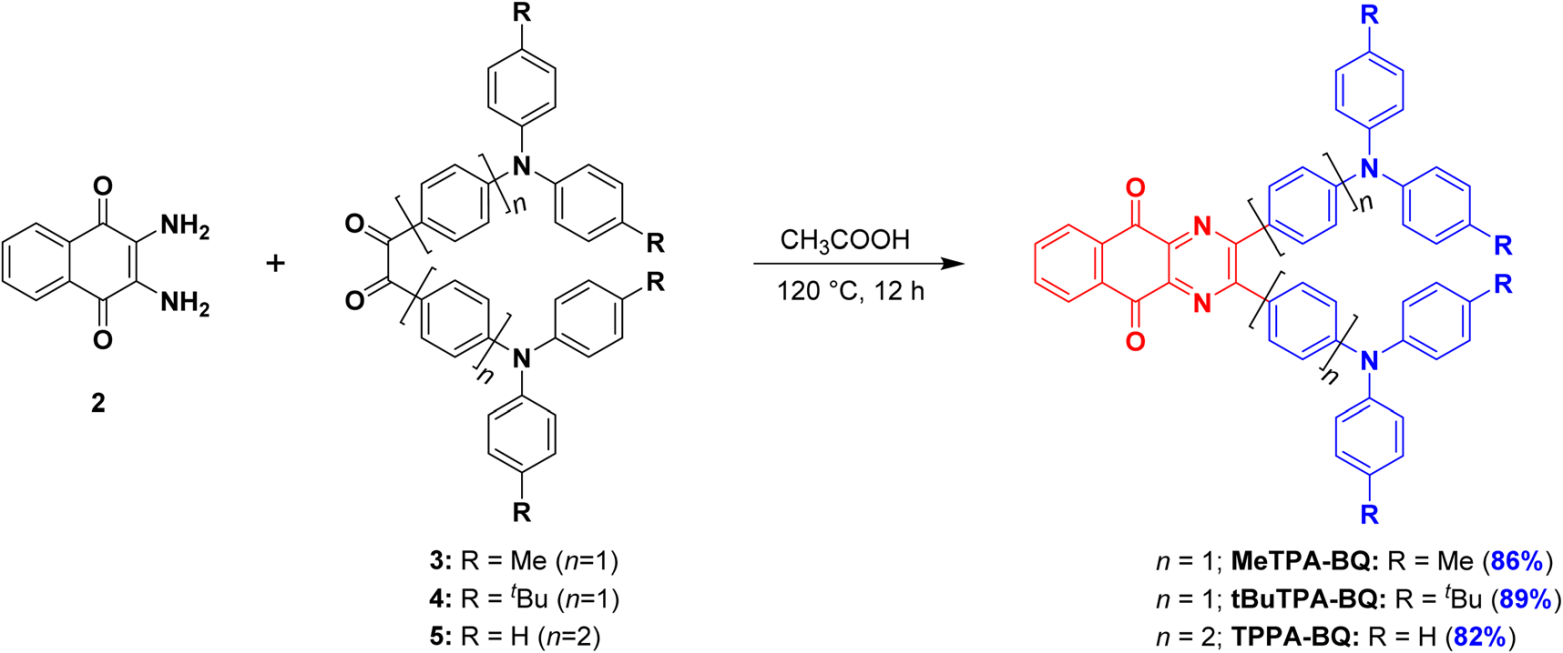
Synthesis of target emitters.

The syntheses of the three target emitters are shown in Scheme 1. The key acceptor intermediate 2 was prepared in two steps with an overall yield of 85%, involving the reaction of 2,3-dichloro-1,4-naphthoquinone with potassium phthalide to furnish 1, which was then subjected to hydrazine hydrate (Scheme S1†). Precursors 3 and 4 are prepared by Buchwald-Hartwig cross-coupling reaction of 4,4′-dibromobenzil with di-*p*-tolylamine and bis(4-(*tert*-butyl)phenyl)amine in 60 and 74% yields, respectively. A Suzuki–Miyaura cross-coupling reaction between 4,4′-dibromobenzil and triphenylamine-4-boronic acid afforded 5 in 81% yield. Target emitters MeTPA-BQ, tBuTPA-BQ and TPPA-BQ were prepared from the condensation of 2 with precursors 3, 4 and 5, respectively, in good yields. The identity and purity of the compounds are confirmed by NMR (^1^H and ^13^C), high-resolution mass spectrometry (HRMS) and high-pressure liquid chromatography (HPLC) and elemental analysis (Fig. S2–S19†).

**Fig. 2:**
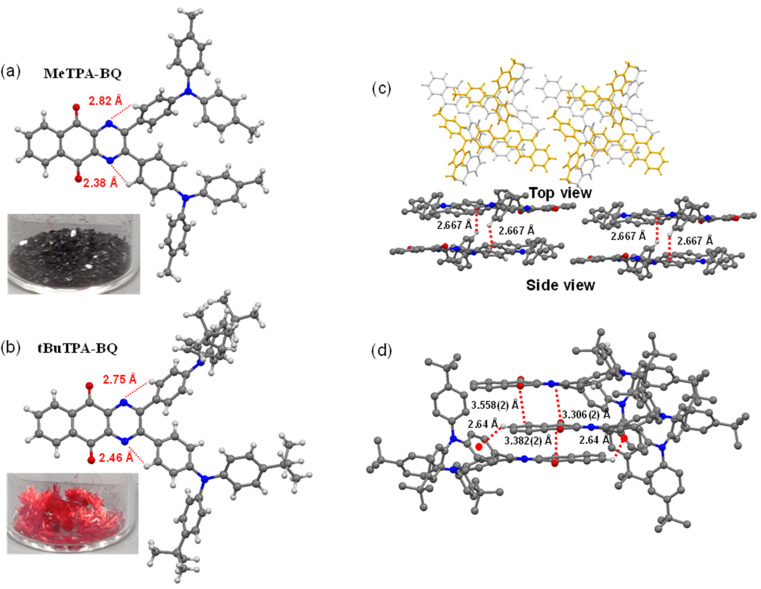
Molecular structures of (a) MeTPA-BQ and (c) interactions between adjacent molecules of MeTPA-BQ (only those H atoms involved in interactions are shown); (b) the molecular structure of tBuTPA-BQ and (d) interactions between adjacent molecules of tBuTPA-BQ (only those H atoms involved in interactions are shown). Red dots (⋯) indicate electrostatic interactions (the inset photos are crystals under daylight), minor components of disorder are omitted.

Single crystals of MeTPA-BQ and tBuTPA-BQ were obtained from the slow evaporation of dichloromethane solution and temperature-gradient vacuum sublimation, respectively. Attempts to grow crystals of TPPA-BQ were unsuccessful. Both MeTPA-BQ and tBuTPA-BQ crystallized in the triclinic space group *P*1̄ (Fig. 2a and b, Table S1†). For MeTPA-BQ, the torsion angles of 58.44(16)° and 22.63(16)° between the acceptor and the phenylene rings are similarly twisted to those in tBuTPA-BQ [(51.9(3)° and 21.5(3)°, respectively]. The smaller torsion for one of the rings is due to the strong intramolecular C–H⋯N hydrogen bonding interactions (H⋯A distances of 2.38 Å for MeTPA-BQ and 2.46 Å for tBuTPA-BQ) between the nitrogen on quinoxaline and the *ortho* C–H of the attached phenyl, enforcing the quasi-planarity. There are weak C–H⋯π interactions between the molecules of MeTPA-BQ in a head-to-tail molecular arrangement in the crystal packing (H⋯centroid distance of 2.68 Å, Fig. 2c). In contrast, there are mutually supporting C=O⋯π (O⋯centroid distances 3.306(2), 3.3819(19), and 3.558(2) Å) and C–H⋯π (H⋯centroid distance of 2.64 Å) contacts between neighboring molecules of tBuTPA-BQ in the crystal lattice (Fig. 2d).

**Fig. 3:**
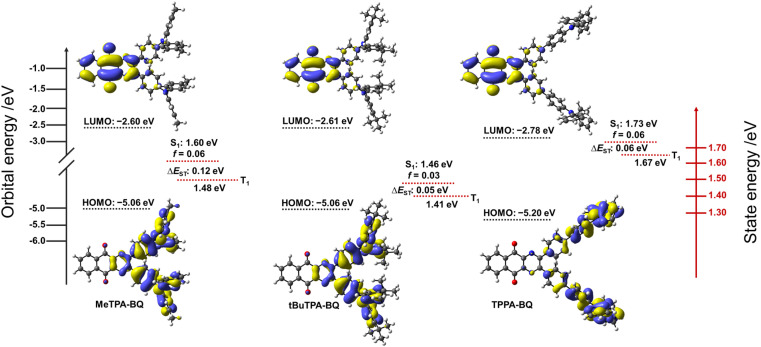
Theoretical modeling of the energies and electron density distributions of the HOMO/LUMO orbitals (ISO value = 0.02) computed based on the ground state S_0_ geometries and their S_1_ and T_1_ energies of MeTPA-BQ, tBuTPA-BQ and TPPA-BQ computed based on the excited state S_1_ geometries.

Density Functional Theory (DFT) calculations at the PBE0 ^17^/6-31G(d,p)^18^ level of theory were performed in the gas phase to provide insight into the electronic structure of the molecules (Fig. 3). The excited-state properties were calculated using time-dependent density functional theory (TD-DFT) within the Tamm–Dancoff approximation (TDA-DFT)^19^ based on the optimized excited S_1_ state geometries.

In the case of MeTPA-BQ and tBuTPA-BQ, the HOMO is located on the triphenylamine (TPA) donor moieties along with some contribution from the pyrazine, while the LUMO is located on the benzo[g]quinoxaline-5,10-dione (BQ) acceptor. In the case of TPPA-BQ, the HOMO is delocalized across both the TPA and the adjacent phenylene spacer and there is no electron density on the pyrazine, while the LUMO is localized on the BQ acceptor. As a result, the HOMO/LUMO energy levels are stabilized in TPPA-BQ (−5.20/−2.78 eV) in comparison to both MeTPA-BQ (−5.06/−2.60 eV) and tBuTPA-BQ (−5.06/−2.61 eV). The computed HOMO–LUMO gaps for MeTPA-BQ, tBuTPA-BQ and TPPA-BQ are 2.46, 2.45 and 2.42 eV, respectively, which are nearly the same, indicating that there is no significant impact of either the donor strength or spacer length on HOMO–LUMO gaps. The S_1_ and T_1_ energies of TPPA-BQ are 1.73 and 1.67 eV, respectively, which are stabilized to 1.60 and 1.48 eV for MeTPA-BQ and 1.46 and 1.41 eV for tBuTPA-BQ. The calculated Δ*E*_ST_ values for TPPA-BQ (0.06 eV) and tBuTPA-BQ (0.05 eV) are smaller than that of MeTPA-BQ (0.12 eV). Natural transition orbital (NTO) analysis based on the S_1_-optimized geometry revealed that the hole is located on the TPA segments, and the particle is located on the BQ acceptor for the S_1_ and T_1_ states of the MeTPA-BQ (Table S2†). Similarly, in the case of tBuTPA-BQ and TPPA-BQ, the hole is mostly located on one TPA group with a very small amount on the other TPA, while the particle is located on the BQ. Nearly similar electron distribution patterns at their S_1_ and T_1_ states resulted in small spin–orbit coupling matrix elements (〈S_1_|*Ĥ*_SOC_|T_1_〉 for MeTPA-BQ (0.13 cm^−1^), tBuTPA-BQ (0.06 cm^−1^) and TPPA-BQ (0.03 cm^−1^) in the gas phase. Thus, relatively slow *k*_RISC_ from T_1_ to S_1_ is expected in all three compounds.

### Optoelectronic studies

**Fig. 4:**
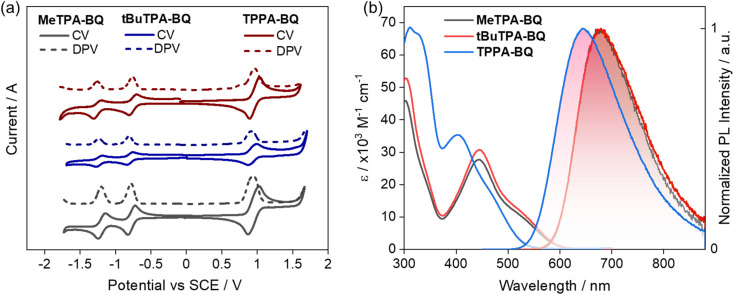
(a) Cyclic voltammograms (CV) and differential scanning calorimetry (DPV) of MeTPA-BQ, tBuTPA-BQ, and TPPA-BQ in N_2_-saturated DCM solution with 0.1 M [^*n*^Bu_4_N]PF_6_ as the supporting electrolyte and Fc/Fc^+^ as the internal reference (0.46 V for DCM *vs.* SCE)^22^ and a scan rate of 100 mV s^−1^. (b) Absorption and emission spectra of MeTPA-BQ, tBuTPA-BQ and TPPA-BQ in the toluene (*λ*_exc_ = 460 nm).

The experimental HOMO and LUMO energies were estimated using cyclic and differential pulse voltammetry (CV and DPV) in DCM with 0.1 M tetra-*n*-butylammonium hexafluorophosphate as the supporting electrolyte (Fig. 4a). The electrochemical data were referenced to Fc/Fc^+^ and are reported relative to a saturated calomel electrode (SCE). Both compounds show two highly reversible reduction waves, typical behavior of the benzoquinone moiety.^20^ The first reduction potentials are −0.78, −0.79 and −0.76 V for MeTPA-BQ, tBuTPA-BQ and TPPA-BQ respectively. The respective estimated LUMO energy levels are −3.56, −3.55 and −3.58 eV, following the trends predicted by DFT calculations that the LUMO energy level of TPPA-BQ is stabilized in comparison to MeTPA-BQ and tBuTPA-BQ (Table S2†). The oxidation potentials were also found to be reversible, with *E*_ox_ of 0.94, 0.91 and 0.97 V for MeTPA-BQ, tBuTPA-BQ and TPPA-BQ, respectively, reflecting the relative donor strength of the substituted TPA (*tert*-butyl > Me > H). The corresponding HOMO energy levels are −5.28, −5.25 and −5.31 eV, indicating that HOMO energy level TPPA-BQ is slightly more stabilized than the other two analogues. Although there is a visible subtle difference in measured HOMO levels between MeTPA-BQ and tBuTPA-BQ, DFT calculations predicted that they be the same. The electrochemical band gap was found to be 1.72, 1.70, and 1.73 eV for MeTPA-BQ, tBuTPA-BQ and TPPA-BQ, respectively. While the absolute values do not correlate with the DFT calculations, the distribution of HOMO–LUMO gaps is well modelled (Table S3†).

The absorption and emission spectra of the three compounds are shown in Fig. 4b. The absorption spectra of MeTPA-BQ and tBuTPA-BQ consist of bands peaking at 302 and 445 nm, along with a broad shoulder at around 535 nm. Not surprisingly, the molar absorptivity (*ε*) values for these bands are very similar for the two compounds given their similar structures. A similar absorption spectral profile was noted for TPPA-BQ; however, the high-energy band at 312 nm is slightly red-shifted while the low energy band and associated shoulder at 405 and 460 nm are blue-shifted compared to the former two compounds. These low-energy bands in all three compounds are due to the charge transfer transitions from the donor fragments to the benzo[*g*]quinoxaline-5,10-dione acceptor moiety, while the short wavelength bands can be ascribed to the donor-centered absorption bands assigned based on comparison with the literature.^21^ The red-shifted CT bands in MeTPA-BQ and tBuTPA-BQ are due to a shorter phenylene spacer that facilitates increased conjugation as illustrated by the DFT-predicted HOMO and LUMO overlap (Fig. 2). The absorption spectra are insensitive to the solvent polarity indicating weak/negligible CT character in the ground state as evidenced by the small, computed ground-state dipole moments of 4.83, 4.66, and 3.16 D for MeTPA-BQ, tBuTPA-BQ and TPPA-BQ, respectively (Fig. S20†).

The photoluminescence spectra in toluene are broad and unstructured, indicating emission from a CT state. MeTPA-BQ and tBuTPA-BQ emit at *λ*_PL_ of 680 nm while TPPA-BQ showed a blue-shifted emission at 645 nm. The PL spectra in different solvents showed positive solvatochromism that corroborates the CT nature of the singlet excited state (Fig. S20†).

**Fig. 5:**
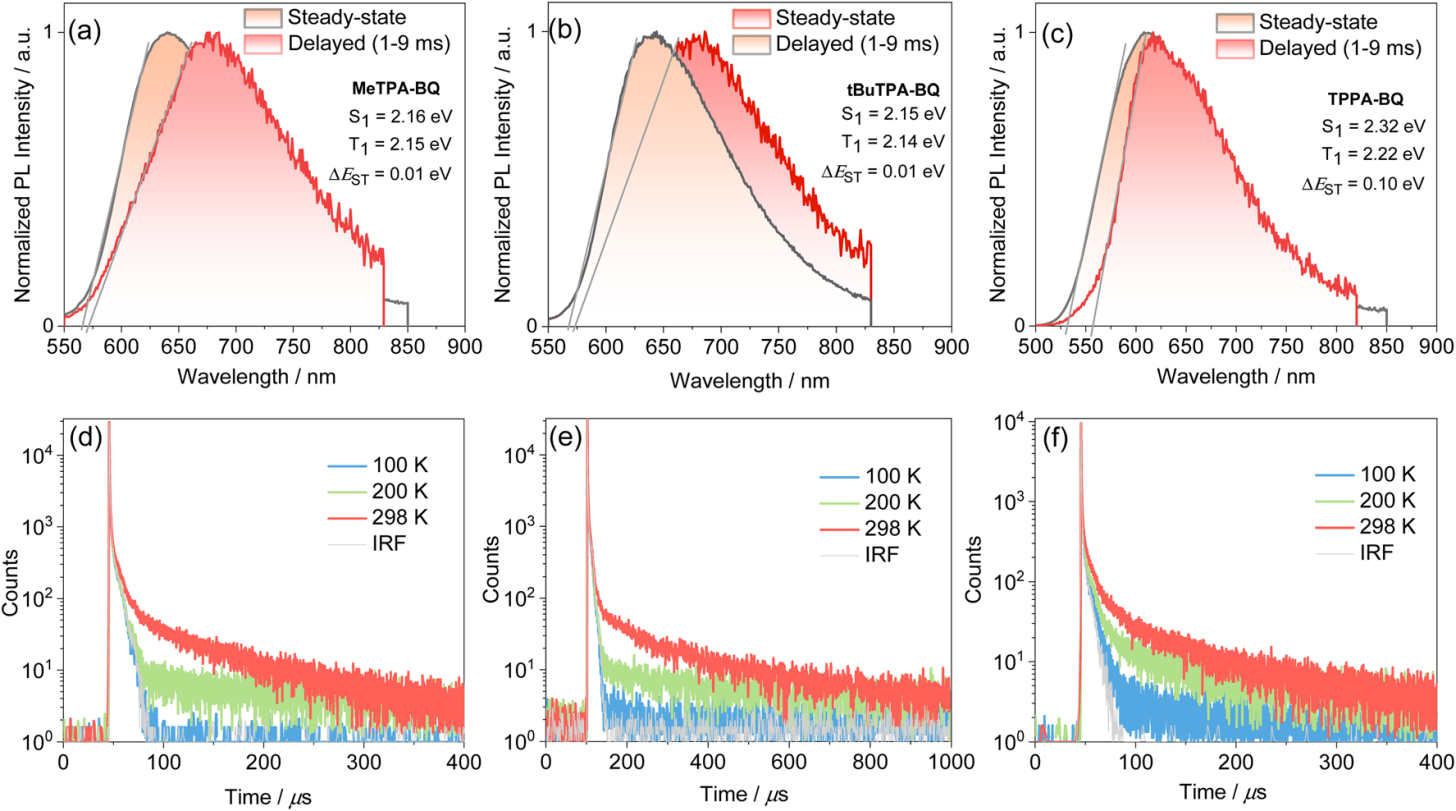
Steady-state PL and phosphorescence spectra (1–9 ms) in 2-MeTHF at 77 K of (a) MeTPA-BQ, and (b) tBuTPA-BQ (c) TPPA-BQ (*λ*_exc_ = 450 nm). Temperature-dependent time-resolved PL decay of (d) MeTPA-BQ, and (e) tBuTPA-BQ (f) TPPA-BQ in 2 wt% doped CBP films (*λ*_exc_ = 340 nm).

The steady-state PL and phosphorescence spectra were measured in 2-MeTHF glass at 77 K and S_1_ and T_1_ energies were determined from their respective onsets (Fig. **5a–c**). The shape of the steady-state and phosphorescence spectra are broad and structureless, illustrating that both states at cryogenic temperatures are CT in nature. The S_1_ and T_1_ energies are 2.16 eV and 2.15 eV for MeTPA-BQ, 2.15 eV and 2.14 eV for tBuTPA-BQ, and 2.32 eV and 2.22 eV for TPPA-BQ. The corresponding Δ*E*_ST_ are 0.01 eV, 0.01 eV, and 0.10 eV. The emissions of MeTPA-BQ, tBuTPA-BQ, and TPPA-BQ in degassed toluene solution decay mono-exponentially, with *τ*_PL_ of 5.4, 5.8 and 3.9 ns, there is no delayed emission observed (Fig. S21†). Next, we investigated the photophysical properties in 4,4′-bis(*N*-carbazolyl)-1,1′-biphenyl (CBP) host as this host matrix has a sufficiently high triplet energy (T_1_ = 2.6 eV) to prevent the backward energy transfer from the T_1_ states of the dopant emitters.^23^ An optimized doing concentration of 2 wt% was identified based on an assessment of the *Φ*_PL_ as a function of dopant concentration from 2–10 wt% in CBP (Table S4†). The MeTPA-BQ, tBuTPA-BQ, and TPPA-BQ emit at *λ*_PL_ of 670, 650, and 625 nm in 2 wt% doped films in CBP, emission that is blue-shifted compared to their respective spectra in toluene solution at *λ*_PL_ of 680, 680 and 645 nm (Fig. S22†). The corresponding film *Φ*_PL_ are 32, 30 and 28% under air and these values increased to 42, 41, and 39% under N_2_. Compounds **MeTPA-BQ, tBuTPA-BQ,** and TPPA-BQ all showed increasing delayed emission with increasing temperature, indicative that these compounds are TADF emitters (Fig. 5). The emissions of MeTPA-BQ, tBuTPA-BQ, and TPPA-BQ decay with multiexponential kinetics, with average prompt lifetimes (*τ*_avg,p_) of 8.0, 9.9 and 12.2 ns, and average delayed lifetimes (*τ*_avg,d_) of 40.1, 144, and 42.8 μs, in 2 wt% doped films in CBP, respectively (Fig. 5, S23, Table S5†). Using these average lifetimes, the *k*_ISC_ and *k*_RISC_ were calculated^24^ to be 2.9 × 10^7^ and 3.3 × 10^4^ s^−1^ for MeTPA-BQ, 2.7 × 10^7^ and 0.9 × 10^4^ s^−1^ for tBuTPA-BQ, and 2.3 × 10^7^ and 3.2 × 10^4^ s^−1^ for TPPA-BQ, respectively, indicating that all three emitters have nearly the same RISC rate constants (Table 1). These *k*_RISC_ values are relatively slower compared to the reported carbonyl-containing quinoxaline acceptor-based TADF emitter (2,3-bis(4-(10*H*-phenoxazin-10-yl)phenyl)quinoxalin-6-yl)(phenyl)methanone, DPXZ-PQM (*k*_RISC_ = 2.05 × 10^5^ s^−1^).^25^ This is due to its higher *Φ*_PL_ of 88% and shorter *τ*_d_ of 3.8 μs due to the smaller Δ*E*_ST_ of 0.02 eV in 5 wt% doped in DCzDPy (5,5′-bis(carbazol-9-yl)-3,3′-bipyridine) films.

**Table 1:** Summary of the photophysical properties of the MeTPA-BQ, tBuTPA-BQ and TPPA-BQ

Emitter	*λ*_abs_ a/nm	*λ*_PL_/nm solution^a^/film^b^	S_1_^c^/eV	T_1_^d^/eV	Δ*E*_ST_^e^/eV	*Φ*_PL_ f (N_2_/O_2_)/(%)	*τ*_p_ g/ns	*τ*_d_ h/μs	*k*_ISC_ i/ × 10^7^ s^−1^	*k*_RISC_ j/ × 10^4^ s^−1^
MeTPA-BQ	302, 445, 535	680/650	2.16	2.15	0.01	42/32	8.0	40.1	2.9	3.3
tBuTPA-BQ	302, 445, 535	680/670	2.15	2.14	0.01	41/30	9.9	144	2.7	0.9
TPPA-BQ	312, 405, 460	645/625	2.32	2.22	0.10	39/28	12.2	42.8	2.3	3.2

a Toluene (*λ*_exc_ = 450 nm).

b 2 wt% doped films in CBP (*λ*_exc_ = 340 nm).

c S_1_ state energy determined from the onset of steady-state PL spectra at 77 K in 2-Me-THF (*λ*_exc_ = 450 nm).

d T_1_ state energy determined from the onset of phosphorescence spectra (1–9 ms) at 77 K in 2-Me-THF (*λ*_exc_ = 450 nm).

e Δ*E*_ST_ = S_1_ – T_1_.

f *Φ*_PL_ was recorded under air/N_2_ atmosphere using an integrating sphere for 2 wt% doped films in CBP (*λ*_exc_ = 340 nm).

g Prompt average lifetime (*τ*_p_) was recorded using time-correlated single photon counting (TCSPC) (*λ*_exc_ = 375 nm) and.

h Delayed average lifetime (*τ*_d_) was recorded using a microsecond flash lamp (*λ*_exc_ = 340 nm).

i *k*_ISC_ = *k*_p_(*Φ*_d_/*Φ*_PL_) – *k*_d_(*Φ*_d_/*Φ*_P_).

j *k*_RISC_ = *k*_d_(*Φ*_PL_/*Φ*_P_). *k*_p_ = 1/*τ*_p_ and *k*_d_ = 1/*τ*_d_. The *k*_p_ and *k*_d_ are the rate constants for the prompt fluorescence and delayed fluorescence, respectively, *k*_ISC_ = intersystem crossing rate constant, *k*_RISC_ = reverse intersystem crossing rate constant, *Φ*_P_ and *Φ*_d_ are the prompt fluorescence and delayed photoluminescence quantum yields.

### Piezochromism

**Fig. 6:**
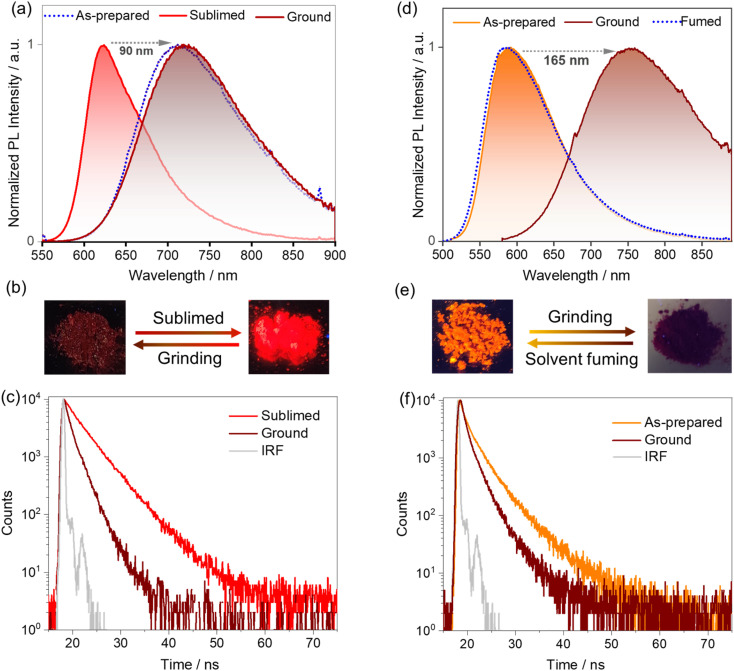
(a) Photoluminescence spectra (*λ*_exc_ = 450 nm) of the as-prepared, sublimed, and ground samples of tBuTPA-BQ; (b) corresponding photographs (under UV torch, *λ*_exc_ = 365 nm) and, (c) time-resolved PL decay (*λ*_exc_ = 375 nm). (d) Photoluminescence spectra (*λ*_exc_ = 450 nm) of the as-prepared, ground, and fumed with EtOAc samples of TPPA-BQ; (e) corresponding photographs (under UV torch, *λ*_exc_ = 365 nm); (f) time-resolved PL decay (*λ*_exc_ = 375 nm).

**Fig. 7:**
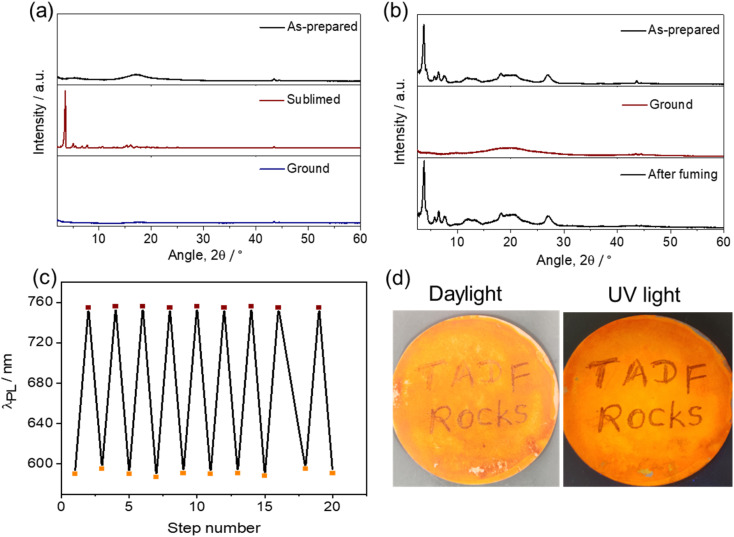
(a) PXRD pattern of the as-prepared, sublimed and ground samples of tBuTPA-BQ, (b) PXRD pattern as-prepared, ground, and fumed with EtOAc samples of TPPA-BQ, (c) repeated switching of the photoluminescence emission wavelength upon mechanical pressure and EtOAc fuming. (d) Demonstration of writing and erasing on filter paper (photos taken under daylight and UV light (*λ*_exc_ = 365 nm).

A change in luminescence upon grinding of tBuTPA-BQ and TPPA-BQ prompted us to investigate the piezochromic properties of these compounds in detail. Both as-prepared and sublimed samples of MeTPA-BQ emit in the near-IR region at *λ*_PL_ of 735 nm. Upon grinding, there is only a modest red-shifting of the emission to 745 nm (Fig. S24†). The as-prepared sample of tBuTPA-BQ emits at *λ*_PL_ = 715 nm (FWHM = 152 nm); however, the emission of the sublimed form is significantly blue-shifted and not as broad (*λ*_PL_ = 622 nm; FWHM = 82 nm) (Fig. 6a), having a *Φ*_PL_ of 10.8% and a *τ*_PL_ of 4 ns. Similar to that observed for MeTPA-BQ, the as-prepared sample of tBuTPA-BQ showed little piezochromism. Surprisingly, the application of mechanical pressure on the sublimed sample results in a significant change in its photophysics, where after grinding, the sample now emits at *λ*_PL_ of 720 nm (FWHM = 148 nm) and has a *Φ*_PL_ of only 0.8% and a *τ*_PL_ of 1.7 ns (Fig. 6b and c). Upon exposure to solvent fumes (hexane, Et_2_O, DCM, THF and EtOAc), the photophysics of tBuTPA-BQ do not revert to the as-prepared form (Fig. S25†). Heating the ground powder to 200 °C also did not result in any change in the photophysics. Powder X-ray diffraction (PXRD) measurements were performed in order to gain deeper insight into the observed mechanochromic behavior of these compounds (Fig. 7a). The PXRD analysis of tBuTPA-BQ showed resolved peaks, indicating the crystalline nature of the sublimed sample. The diffractograms of the ground powder and as-prepared sample did not show any noticeable reflection peaks indicating that these samples are in an amorphous state. These observations demonstrate a morphological transition between the crystalline (ordered) and amorphous (disordered) phases. None of the pure solid samples of tBuTPA-BQ showed TADF as-prepared, sublimed, or ground (Fig. S26†).

The compound TPPA-BQ showed an even more remarkable red-shift of ∼165 nm upon grinding. The as-prepared sample emits at 590 nm (FWHM = 106 nm), has a *Φ*_PL_ of 3% and a *τ*_PL_ of 5.2 ns, while the ground sample emits at 755 nm (FWHM = 195 nm), has a much diminished *Φ*_PL_ of 0.2% and a *τ*_PL_ of 1.8 ns (Fig. 6d–f). Akin to tBuTPA-BQ, the ground form of TPPA-BQ was stable under ambient conditions and the photophysics did not revert to the as-prepared form upon heating to 200 °C. However, the photophysics of the ground form could be converted to that of the as-prepared form upon fuming with EtOAc. Akin to the sublimed form of tBuTPA-BQ, the PXRD of TPPA-BQ showed a resolved diffraction peaks for the as-prepared sample, indicating its crystalline nature (Fig. 7b). The diffractograms of the ground powder did not show any noticeable patterns indicating that these samples are in an amorphous state. The PXRD pattern is perfectly restored after EtOAc fuming of the ground sample of TPPA-BQ (Fig. 7b). The as-prepared sample of TPPA-BQ showed multiexponential emission decay kinetics with *τ*_P_ of 5.2 ns and average *τ*_d_, of 60 μs. Variable temperature time-resolved PL measurements further confirmed the TADF behavior of as-prepared sample of TPPA-BQ (Fig. S27†). However, when ground no TADF was observed, possibly due to its weak PL behavior. The ^1^H NMR spectra of the ground samples dissolved in CDCl_3_ were the same as those of the as-prepared samples, indicating that grinding did not decompose the compounds, and that a solid-state transformation was responsible for the piezochromism.

In the solid-state absorption spectra of TPPA-BQ, distinct differences were observed for the as-prepared and ground samples (Fig. S28†). The as-prepared sample showed absorption bands at ∼310, ∼410 and ∼475 nm. The ground sample has the same ∼310 nm band but there is a new band at ∼420 nm with a tail extending to 600 nm. Such a long wavelength tail likely indicates that there is partial conformational planarization leading to an increase in the conjugation length to some extent in the ground form (Fig. S28†). The as-prepared (crystalline) TPPA-BQ emits at 590 nm, which is close to the emission (600 nm) of the 0.5 wt% doped film in PMMA, clearly indicating that the emission of the as-prepared sample reflects the emission from monomolecular species that is present in the dispersed state. Therefore, we attribute the large spectral shift (165 nm) for TPPA-BQ to emission from an amorphous aggregate (Fig. S29†). The absorption spectra of the sublimed and ground samples of tBuTPA-BQ are similar, indicating that no significant changes occurred to its conformation upon grinding. Similarly, the sublimed sample (crystalline) of tBuTPA-BQ emits at 622 nm, which is similar to the emission (631 nm) of the 0.5 wt% doped film in PMMA, clearly indicating that the emission of the sublimed sample reflects the emission from monomolecular species that is present in the dispersed state. Upon grinding, the emission originates from an amorphous aggregate (Fig. S29†). For MeTPA-BQ, the emission from the as-prepared (crystalline, 735 nm) sample results from an aggregate as it is much different from the emission observed in the 0.5 wt% doped films in PMMA (622 nm). Upon grinding, the emission originated from the amorphous aggregate (745 nm).

The grinding and fuming cycles for TPPA-BQ were repeated 20 times and showed a high degree of reproducibility, confirming the reversibility of the phase transformations and with no obvious fatigue response (Fig. 7c). To demonstrate the practical application of TPPA-BQ as a photoluminescent ink, the compound was dispersed in EtOAc under ultrasonication for 2 min. The dispersed suspension was coated onto a filter paper by drop casting and left to dry for 1 h. A glass rod was used as the writing implements on the substrate (Fig. 7d), and the areas of the filter paper upon which the rod was in contact showed a contrasting reddish color, indicative of the ground form while the rest of the filter paper emitted yellow-orange emission under UV light illumination. The distinct emission color change of the written text remained for at least 2 months of observation. The text could be erased upon the addition of EtOAc drops to the written region, while DCM, hexane and THF did not produce the same behavior.

### Device characterization

**Fig. 8:**
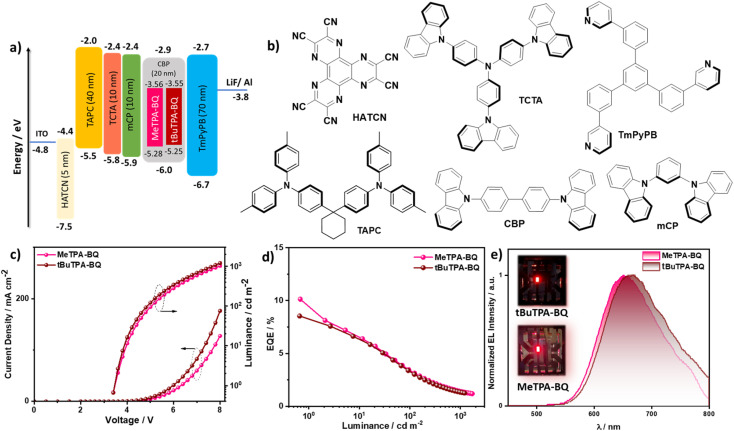
(a) Energy level diagram of materials employed in the devices; (b) molecular structure of materials used in the devices; (c) current density and luminance *versus* voltage characteristics for the devices; (d) external quantum efficiency *versus* luminance curves for the devices; (e) electroluminescence spectra of the devices, the inset is photograph images of the electroluminescence from the devices.

The observed TADF behavior of MeTPA-BQ and tBuTPA-BQ in the deep-red region at 650 and 670 nm in CBP prompted us to explore their use as emitters in OLEDs. We fabricated vacuum-deposited bottom-emitting OLEDs using an optimized device structure of: indium-tin-oxide (ITO)/1,4,5,8,9,11-hexaazatriphenylenehexacarbonitrile (HATCN) (5 nm)/1,1-bis[(di-4-tolylamino)phenyl]cyclohexane (TAPC) (40 nm)/tris(4-carbazoyl-9-ylphenyl)amine (TCTA) (10 nm)/1,3-bis(*N*-carbazolyl)benzene (mCP) (10 nm)/emissive layer (20 nm)/1,3,5-tri[(3-pyridyl)-phen-3-yl]benzene (TmPyPB) (70 nm)/LiF (0.7 nm)/Al (100 nm), where HATCN is the hole-injection layer (HIL), TAPC and TCTA play the role in hole-transporting layers (HTL), mCP acts as an electron-blocking layer (EBL) and TmPyPB played two roles as an electron-transport layer and a hole-blocking layer due to its deep HOMO (−6.7 eV), and LiF^26^ acts as an electron-injection layer from the aluminum cathode. The emitter doping concentration of 2 wt% was selected based on an optimization *Φ*_PL_ study in CBP (*vide supra*). The molecular structures of the materials used in these devices, energy level diagram, the EQE–luminance, current density–voltage–luminance (*J–V–L*) curves, and electroluminescence spectra (EL) are shown in Fig. 8 and the data compiled in Table 2. As shown in Fig. 8e, the devices with MeTPA-BQ and tBuTPA-BQ showed deep-red emission (*λ*_EL_) at 650 and 670 nm with CIE coordinates of (0.645, 0.344) and (0.656, 0.336), respectively, emissions that align with the PL spectra in CBP doped thin films (Fig. S22†). The MeTPA-BQ based device showed a maximum external quantum efficiency (EQE_max_) of 10.1%, while the tBuTPA-BQ based device showed an EQE_max_ of 8.5% (Fig. S30†). The theoretical EQE_max_ for the devices with MeTPA-BQ and tBuTPA-BQ are 10.5 and 10.3%, considering an outcoupling efficiency of *χ*_out_ ≈ 25% based on a presumed isotropic orientation of the transition dipole moments of the emitters. This implies that all triplet excitons are efficiently converted into singlets in both devices. Both devices showed moderate efficiency roll-off, with the EQE at 100 cd m^−2^ of 3.4%, and the EQE at 1000 cd m^−2^ of around 1.4%. The efficiencies of these devices are comparable with other deep-red (*λ*_EL_ = 650**-**670 nm) TADF OLEDs (Table S6†).

**Table 2:** Electroluminescence data for the devices^a^

Emitter	*λ*_EL_ b/nm	FWHM^b^/nm	CIE^b^/(*x*, *y*)	*V*_on_ c/V	CE_max_/cd A^−1^	PE_max_/lm W^−1^	EQE^d^
MeTPA-BQ	650	123	0.64, 0.34	3.4	5.88	5.43	10.1/3.4/1.4
tBuTPA-BQ	670	134	0.66, 0.34	3.4	4.69	4.33	8.5/3.4/1.3

a Device stack; ITO/HATCN (5 nm)/TAPC (40 nm)/TCTA (10 nm)/mCP(10 nm)/emissive layer (2 wt% emitter in CBP, 20 nm)/TmPyPB (70 nm)/LiF (0.7 nm)/Al (100 nm).

b The electroluminescence maximum, CIE coordinates and FWHM of the EL spectrum recorded at 5 V.

c The turn-on voltage at EQE_max_.

d The order of measured values: the EQE_max_/EQE_100_/EQE_1000_.

## Conclusions

We have developed multi-functional compounds that exhibit both TADF and piezochromism. Despite their similar chemical structures, MeTPA-BQ and tBuTPA-BQ possess distinctly different photophysical properties in the crystalline states, which are attributed to their different solid-state packing. Notably, TPPA-BQ represents the first example of a TADF material that displays such a large piezochromic shift of 165 nm, into the near-infrared region. The changes in the photophysical properties of TPPA-BQ were found to be completely reversible upon grinding and ethyl acetate solvent fuming. MeTPA-BQ and tBuTPA-BQ were employed as emitters in the fabrication of deep red OLEDs showing EQE_max_ of 10.1% (*λ*_EL_ = 650 nm) and 8.5% (*λ*_EL_ = 670 nm), respectively.

## Data availability

The research data supporting this publication can be accessed at https://doi.org/10.17630/7183d304-4115-4dd8-9c7b-373637da4249.

## Author contributions

Project designed by E. Z.-C. and P. S. Synthesis and optoelectronic characterization by P. S. Crystal structure determination by D. B. C. OLED fabrication by A. K. G. All authors contributed to the writing of the manuscript.

## Conflicts of interest

There are no conflicts to declare.

## Supplementary Material

SC-015-D3SC05188A-s001

SC-015-D3SC05188A-s002

SC-015-D3SC05188A-s003
