# Supplementary material for: Thermally activated delayed fluorescence emitters showing wide-range near-infrared piezochromism and their use in deep-red OLEDs
Source: Chem Sci. 2023 Nov 29;15(2):545–54. doi: 10.1039/d3sc05188a (PMC10763033; doi:10.1039/d3sc05188a)
Supplement: SC-015-D3SC05188A-s001 [file SC-015-D3SC05188A-s001.pdf]

Electronic Supplementary Material (ESI) for Chemical Science.
This journal is © The Royal Society of Chemistry 2023

Thermally Activated Delayed Fluorescence Emitters for Wide-Range Near-infrared Piezochromism and Deep-Red OLEDs

*Pagidi Sudhakar, Abhishek Kumar Gupta, David Cordes and Eli Zysman-Colman**

[a]Organic Semiconductor Centre, EaStCHEM School of Chemistry, University of St Andrews, St Andrews, UK, KY16 9ST. E-mail: eli.zysman-colman@st-andrews.ac.uk

Table of contents

Section	Pages
General experimental details…………………………………………………....	S2-S5
Literature reports and reaction scheme……………………………………….	S6-S7
Synthesis and spectral characterization……………………………………....	S8-S20
X-ray crystallography and DFT results……………………………………	S21-S22
Photophysical data………………………………………………………....	S23-S26
OLED data………………………………………………………………	S26-S27
References……………………………………………………………….	S28-S30

Experimental Section

General Synthetic Procedures. The following starting materials were synthesized according to literature materials: 1,2-bis(4-(di-*p*-tolylamino)phenyl)ethane-1,2-dione (**1**),[1] 1,2-bis(4'-(diphenylamino)-[1,1'-biphenyl]-4-yl)ethane-1,2-dione(**2**),[2] 2,2'-(1,4-dioxo-1,4-dihydronaphthalene-2,3-diyl)bis(isoindoline-1,3-dione) (**3**),[3] 2,3-diaminonaphthalene-1,4-dione (**4**).[3] All other reagents and solvents were obtained from commercial sources and used as received. Air-sensitive reactions were performed under a nitrogen atmosphere using Schlenk techniques, no special precautions were taken to exclude air or moisture during work-up and crystallization. Anhydrous tetrahydrofuran, acetonitrile, and toluene were obtained from an MBraun SPS5 solvent purification system. Flash column chromatography was carried out using silica gel (Silica-P from Silicycle, 60 Å, 40-63 μm). Analytical thin-layer-chromatography (TLC) was performed with silica plates with aluminum backings (250 μm with F-254 indicator). TLC visualization was accomplished by 254/365 nm UV lamp. HPLC analysis was conducted on a Shimadzu LC-40 HPLC system. HPLC traces were performed using an ACE Excel 2 C18 analytical column. HPLC and gel permeation chromatography (GPC) was conducted on a Shimadzu LC-40 HPLC system. GPC trace was performed using a Shim-pack GPC-803 column with THF as mobile phase. ^{1}H, and ^{13}C spectra were recorded on a Bruker Advance spectrometer (400 MHz for ^{1}H, 101 or 125 MHz for ^{13}C). The following abbreviations have been used for multiplicity assignments: "s" for singlet, "d" for doublet, "t" for triplet, "dd" for doublet of doublets and "m" for multiplet. ^{1}H and ^{13}C NMR spectra were referenced residual solvent peaks with respect to TMS ($\delta = 0$ ppm). Melting points were measured using open-ended capillaries on an Electrothermal 1101D Mel-Temp apparatus and are uncorrected. High-resolution mass spectrometry (HRMS) was performed at the University of Edinburgh. Elemental analyses were performed by the School of Geosciences at the University of Edinburgh. PXRD was performed at room temperature using a PANalytical Empyrean diffractometer in Bragg-Brentano geometry using Cu Kα1 radiation with a primary beam monochromator ($\lambda = 1.54060$ Å).

Theoretical Calculations. All ground-state optimizations have been carried out at the Density Functional Theory (DFT) level with Gaussian16[4] using the PBE0 functional[5] and the 6-31G(d,p) basis set[6]. Excited-state calculations have been performed at Time-Dependent DFT (TD-DFT)

within the Tamm-Dancoff approximation (TDA)[7] using the same functional and basis set as for ground state geometry optimization. Spin-orbit coupling matrix elements (ξ) were calculated based on the optimized singlet excited state geometry. Molecular orbitals were visualized using GaussView 6.0[8]. Calculations were automated using an in-house designed software package, *Silico*, which uses a number of 3rd party libraries and programs, including extraction and processing of results: cclib[9], generations of 3D images[10]: VMD & Tachyon[11].

Electrochemistry measurements. Cyclic Voltammetry (CV) analysis was performed on an Electrochemical Analyzer potentiostat model 620E from CH Instruments at a sweep rate of 100 mV/s. Differential pulse voltammetry (DPV) was conducted with an increment potential of 0.004 V and pulse amplitude, width, and period of 50 mV, 0.05, and 0.5 s, respectively. Samples were prepared as DCM solutions, which were degassed by sparging with MeCN-saturated argon gas for 5 minutes prior to measurements. All measurements were performed using 0.1 M DCM solution of tetra-*n*-butylammonium hexafluorophosphate ([^{n}Bu$_4$N]PF$_6$]). An Ag/Ag$^+$ electrode was used as the reference electrode while a platinum electrode and a platinum wire were used as the working electrode and counter electrode, respectively. The redox potentials are reported relative to a saturated calomel electrode (SCE) with a ferrocenium/ferrocene (Fc/Fc$^+$) redox couple as the internal standard (0.46 V vs SCE)[12].

Photophysical measurements. Optically dilute solutions of concentrations on the order of 10^{-5} or 10^{-6} M were prepared in spectroscopic or HPLC grade solvents for absorption and emission analysis. Absorption spectra were recorded at room temperature on a Shimadzu UV-2600 double beam spectrophotometer with a 1 cm quartz cuvette. Molar absorptivity determination was verified by linear regression analysis of values obtained from at least four independent solutions at varying concentrations with absorbance ranging from 0.48 to 0.17 for **MeTPA-BQ**, 0.15 to 0.37 for **tBuTPA-BQ** and 0.54 to 0.19 for **TPPA-BQ**.

Toluene solutions were degassed via three freeze-pump-thaw cycles. Steady-state emission and time-resolved emission spectra were recorded at 298 K using an Edinburgh Instruments FS5 fluorimeter. Thin films were then spin-coated on a quartz substrate using a spin speed of 1500 rpm for 60 s. Absolute photoluminescence quantum yields (Φ_{PL}s) were determined using an integrating sphere that is equipped with an FS5 spectrometer. The Φ_{PL}s were measured in air and N_2

environment by purging the integrating sphere with N_2 gas flow for 2 min. Steady-state PL spectra were measured using a xenon lamp as the source. Time-gated PL spectra (delayed emission/phosphorescence) were measured using a pulsed microsecond flash lamp by the multi-channel scaling (MCS) mode in FS5. The time-gated PL spectra for the samples were collected between 1-9 ms (λ_{exc} = 450 nm). Temperature-dependent (100 to 298 K) measurements were performed using an Oxford Instruments OPTISTAT DN-V cryostat controlled by an Oxford Instruments Mercury iTC temperature controller connected to the FS5 spectrometer. Samples were allowed to equilibrate at each temperature before measurements were conducted.

The singlet-triplet energy splitting (ΔE_{ST}) in 2-MeTHF was estimated from the onset of steady-state and phosphorescence emission at 77 K (λ_{exc} = 450 nm). Phosphorescence spectra collected by using pulsed microsecond flashlamp (1-9 ms). Prompt fluorescence lifetimes were measured using a picosecond pulsed diode laser (375 nm). Phosphorescence lifetimes were measured using a pulsed xenon microsecond flash lamp. Samples were ground mechanically using a mortar and pestle for 5 min. Steady-state PL (λ_{exc} = 450 nm) and time-resolved measurements, prompt lifetime using TCSPC (λ_{exc} = 375 nm) and delayed lifetime using microsecond flash lamp (λ_{exc} = 340 nm), were conducted for the as-prepared and ground samples.

Fitting of time-resolved luminescence measurements: Time-resolved PL measurements were fitted to a sum of exponentials decay model, with chi-squared (χ^2) values between 1 and 2, using the EI FLS980 software. Each component of the decay is assigned a weight, (w_i), which is the contribution of the emission from each component to the total emission.

The average lifetime was then calculated using the following:

- Two exponential decay model:

$$\tau_{AVG} = \tau_1 w_1 + \tau_2 w_2$$

with weights defined as $w_1 = \frac{A1\tau_1}{A1\tau_1 + A2\tau_2}$ and $w_2 = \frac{A2\tau_2}{A1\tau_1 + A2\tau_2}$ where A1 and A2 are the preexponential-factors of each component.

- Three exponential decay model:

$$\tau_{AVG} = \tau_1 w_1 + \tau_2 w_2 + \tau_3 w_3$$

with weights defined as $w_1 = \frac{A1\tau_1}{A1\tau_1 + A2\tau_2 + A3\tau_3}$, $w_2 = \frac{A2\tau_2}{A1\tau_1 + A2\tau_2 + A3\tau_3}$ and $w_3 = \frac{A3\tau_3}{A1\tau_1 + A2\tau_2 + A3\tau_3}$ where A1, A2 and A3 are the preexponential-factors of each component.

OLED fabrication and characterization

The OLED devices were fabricated in a bottom-emitting structure via thermal evaporation in a high vacuum at a base pressure of < 5 × 10^{-7} mbar. A pre-patterned glass substrate coated with indium-doped tin oxide (ITO) was cleaned sequentially by ultrasonication in acetone and isopropanol for 15 minutes. The temperature of the ultrasonication bath was set at 60-70 °C. The cleaned substrate was exposed to oxygen plasma for 3 min to remove all dust and organics on the ITO surface and to increase the work function of the ITO anode for better hole injection from the anode to the organic layer. The substrate was loaded in the thermal evaporator. The organic layers were deposited at a rate of 0.3-1.0 Å/s, monitored using a quartz crystal. The electron injection layer, LiF, was deposited at a rate of 0.05 Å/s, while the Al cathode was deposited initially with a rate of 0.5 Å/s to obtain 10 nm thickness and after that, the rate of the Al cathode was increased to 3 Å/s. Two custom-made shadow masks were used to define the area of the evaporations. The organic layers and LiF were evaporated with the same shadow mask, but Al was evaporated with the other mask. The active area of the OLED was 2 mm^2, determined by the spatial overlap of the anode and cathode electrodes. All the devices were encapsulated with glass lids and UV epoxy resin inside a N_2-filled globe box. The luminance-current-voltage characteristics were measured in an ambient environment using a Keithley 2400 source meter and a homemade photodiode circuit connected to a Keithley 2000 multimeter for the voltage reading. The external quantum efficiency was calculated assuming the Lambertian emission pattern for the OLEDs. The electroluminescence spectra were recorded by an Andor DV420-BV CCD spectrometer.

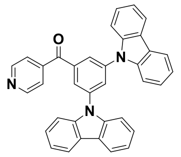

mDCBP

λ_P = 460 nm
λ_G = 500 nm

J. Mater. Chem. C, *2016*, 4, 900

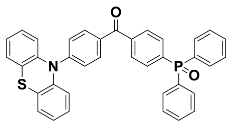

CPzPO

λ_P = 459, 564 nm
λ_G = 564 nm

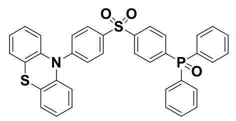

SPzPO

λ_P = 433 nm, 546 nm
λ_G = 546 nm

Adv. Funct. Mater. **2017**, *27*, 1703918

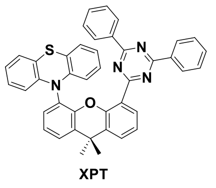

XPT

λ_P = 536 nm
λ_G = 569 nm

J. Am. Chem. Soc., **2017**, *139*, 4894

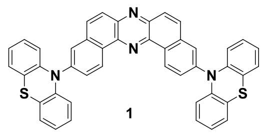

1

λ_P(yellow crystals) = 536 nm
λ_P(orange crystals) = 640 nm
λ_G = 673 nm

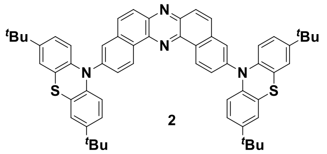

2

λ_P = 547 nm
λ_G = 663 nm

Chem. Sci. **2017**, *8*, 2677

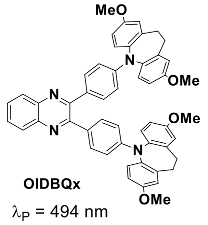

OIDBQx

λ_P = 494 nm
λ_G = 522 nm

Chem. Commun., **2018**, *54*, 13857

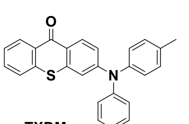

TXDM

λ_P = 470 nm
λ_G = 486 nm

J. Mater. Chem. C,. **2019**, *7*, 3522

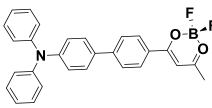

1

λ_P = 596 nm
λ_G = 670 nm

Mater. Chem. Front., **2020**, *4*, 285

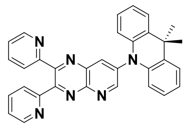

DPP-DMAC

λ_P = 554 nm
λ_G = 608 nm

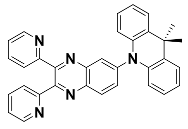

DPQ-DMAC

λ_P = 548 nm
λ_G = 571 nm

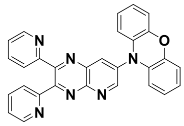

DPP-PXZ

λ_P = 628 nm
λ_G = 682 nm

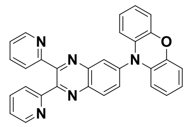

DPQ-PXZ

λ_P = 589 nm
λ_G = 616 nm

Chem. Eng. J. **2020**, *390*, 124626

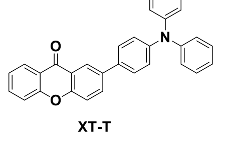

XT-T

λ_P = 478 nm
λ_G = 510 nm

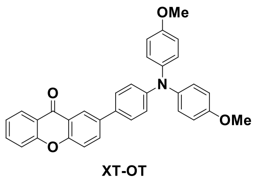

XT-OT

λ_P = 466 nm
λ_G = 567 nm

Dyes Pigm,. **2022,** *205*, 110550

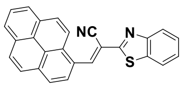

Py-BZTCN

λ_P = 581 nm
λ_G = 676 nm

CCS Chem. **2022**, *4*, 625

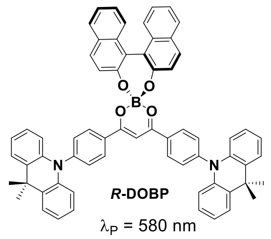

***R*-DOBP**

λ_P = 580 nm
λ_G = 647 nm

Angew. Chem. Int. Ed. **2022**, *61*, 202203844

Figure **S1**. Reported TADF-MCL materials (λ_P and λ_G for pristine and ground emission wavelength, respectively).

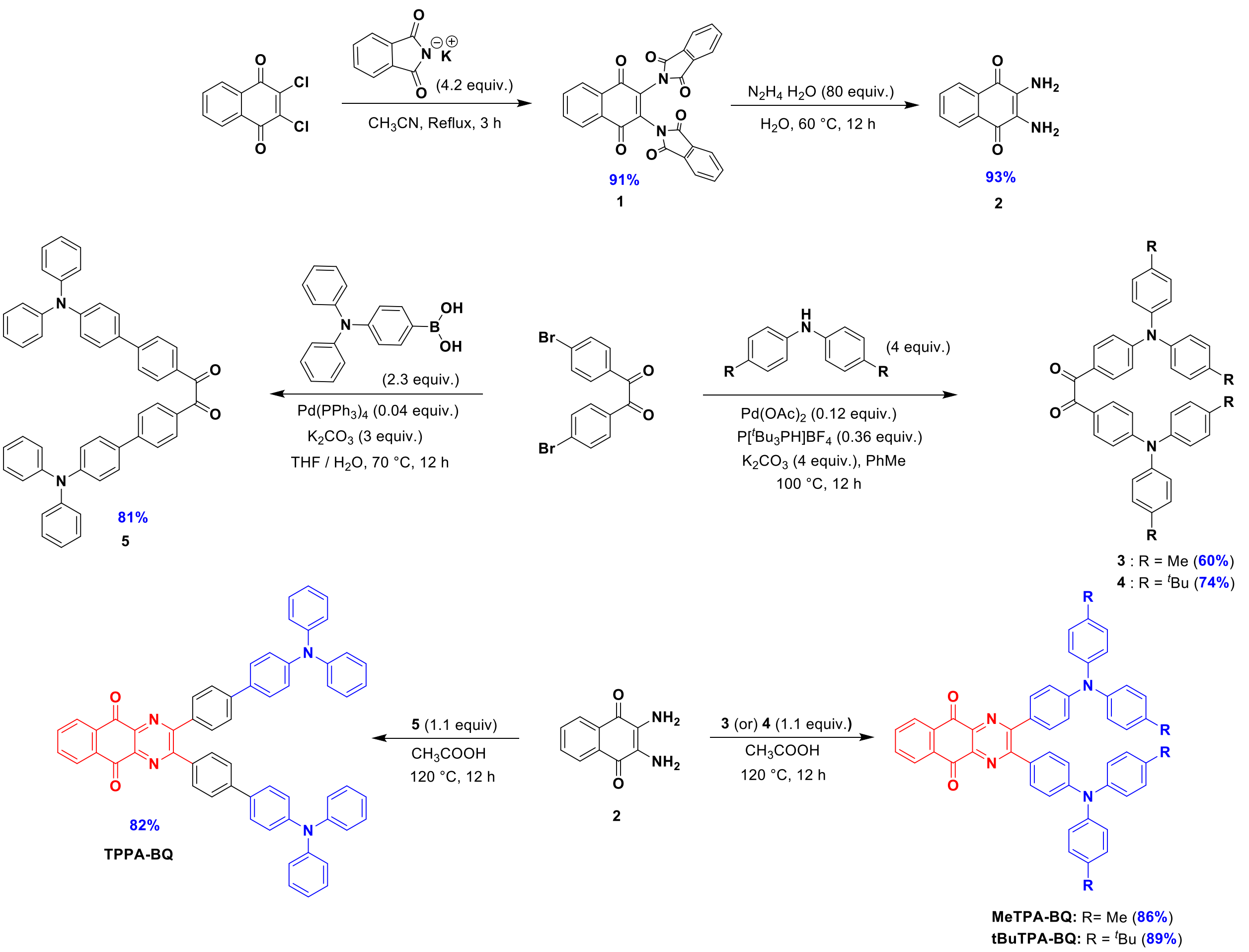

Scheme **S1**. Synthesis of **MeTPA-BQ**, **tBuTPA-BQ** and **TPPA-BQ**

2,3-bis(4-(di-*p*-tolylamino)phenyl)benzo[g]quinoxaline-5,10-dione (MeTPA-BQ)

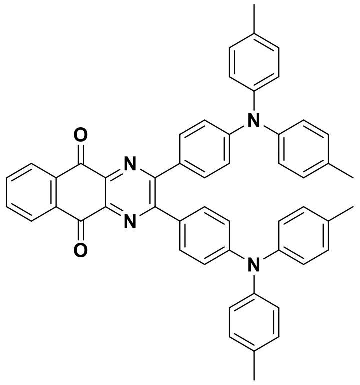

A solution of 2,3-diamino-1,4-naphthoquinone (**2**) (100 mg, 531 μmol, 1 equiv.) and 1,2-bis(4-(di-*p*-tolylamino)phenyl)ethane-1,2-dione (**3**) (351 mg; 584 μmol, 1.1 equiv.) in glacial acetic acid (5 mL) was heated at 100 °C for 3 h. The reaction mixture was allowed to cool to room temperature. The precipitate was filtered, then washed with water. The crude mixture was purified by silica gel flash column chromatography using DCM : hexane = 3:1 as the eluent to afford the desired compound as a white solid. **Yield**: 86%. **R_f:** 0.28 (DCM : hexane = 1:2 on silica gel). **Mp**: 328-330 °C. **^{1}H NMR (400 MHz, $CDCl_3$) δ (ppm)**: 8.40 (dd, *J* = 5.8, 3.3 Hz, 2H), 7.86 (dd, *J* = 5.8, 3.3 Hz, 2H), 7.59 (d, *J* = 8.9 Hz, 4H), 7.26 (s, 1H), 7.11 (d, *J* = 8.1 Hz, 8H), 7.04 (d, *J* = 8.4 Hz, 8H), 6.93 (d, *J* = 8.9 Hz, 4H), 2.35 (s, 12H). **^{13}C NMR (125 MHz, $CDCl_3$) δ (ppm)**: 181.57, 156.09, 150.09, 144.32, 141.22, 134.61, 133.83, 133.31, 131.00, 130.10, 129.18, 127.62, 125.69, 77.37, 77.05, 76.73, 20.96. **HR-MS[M+H]$^+$ Calculated: (**$C_{52}H_{41}N_4O_2$) 753.3224; **Found**: 753.3184. **Anal**. Calcd. for $C_{52}H_{40}N_4O_2$: C, 82.95%; H, 5.36%; N, 7.44%. Found: C, 83.34%; H, 5.54%; N, 7.52%. **HPLC-GPC** purity: 100%. Retention time: 13.2 min.

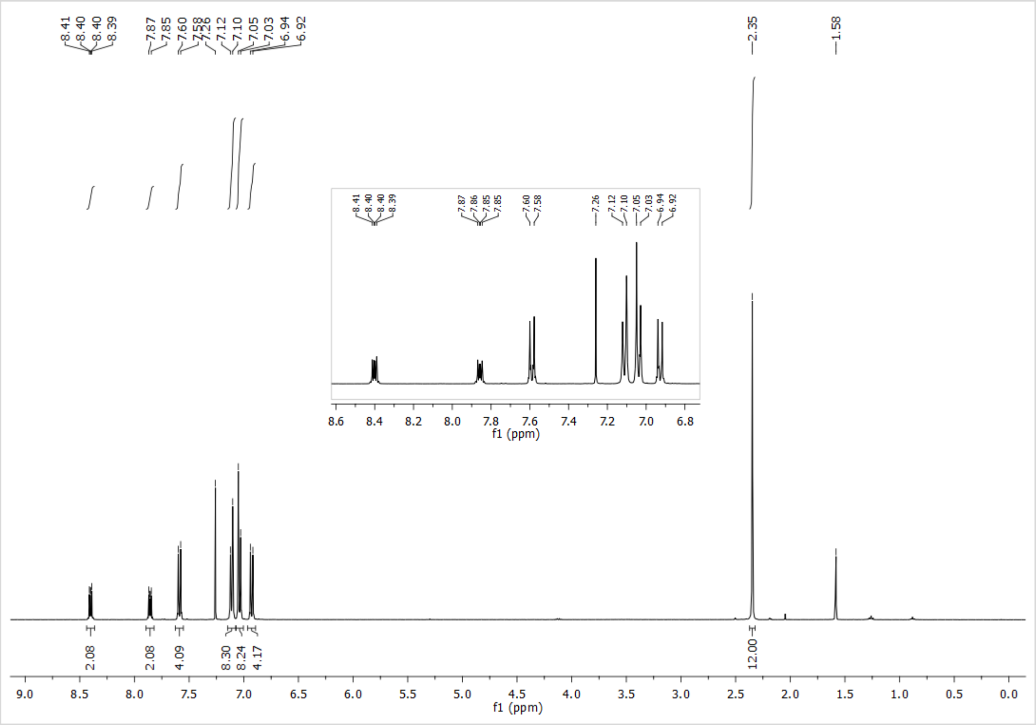

Figure **S2**. ^{1}H NMR of **MeTPA-BQ** in $CDCl_3$

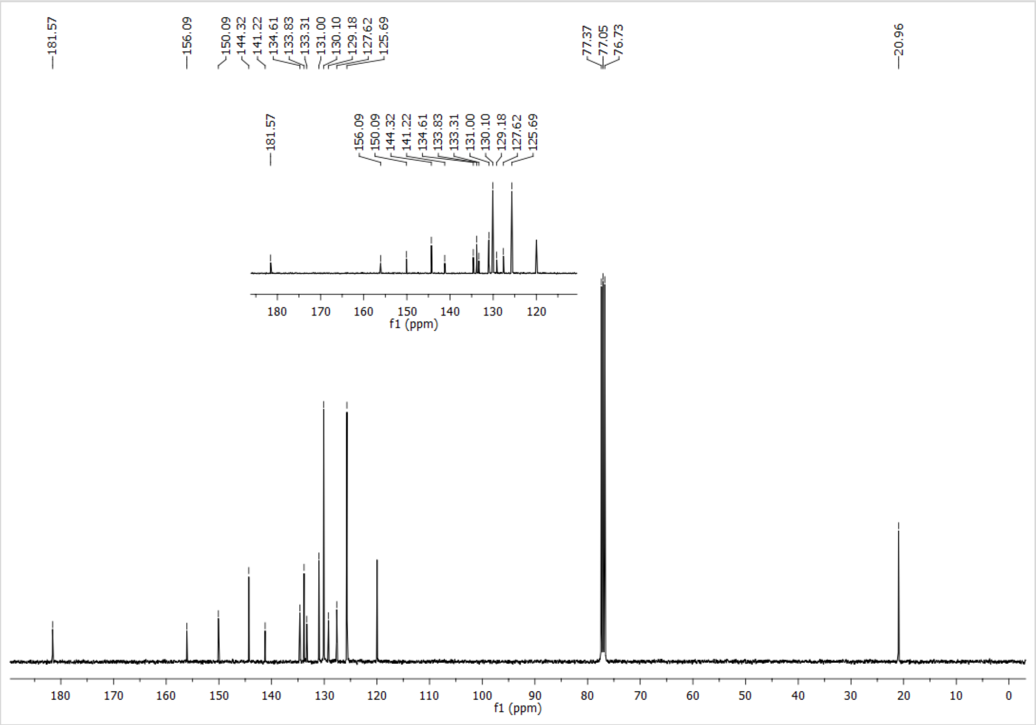

Figure **S3.** ^{13}C NMR of **MeTPA-BQ** in $CDCl_3$

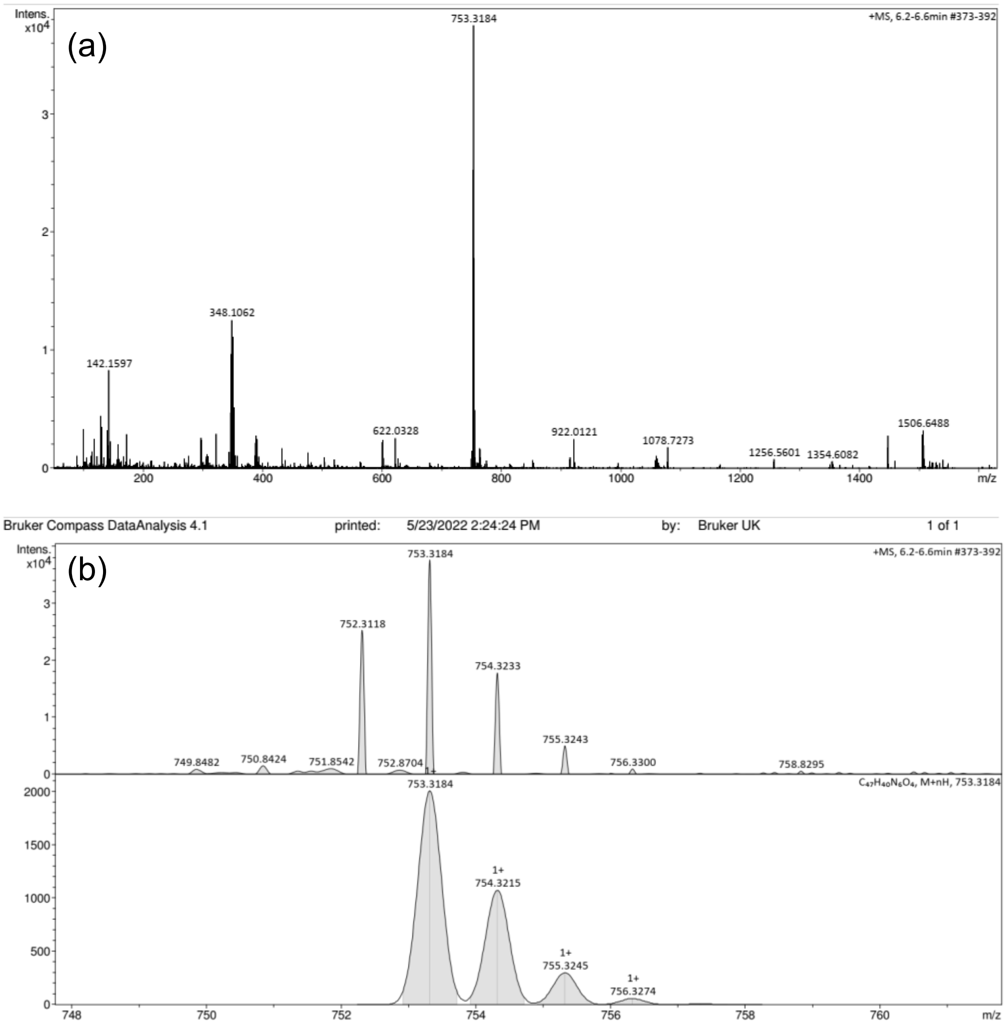

Figure **S4.** HRMS of (a) **MeTPA-BQ** and (b) its isotopic distribution pattern

Analysis Result:

Element	Expected %	Found (1)	Found (2)	Found (3)
Nitrogen	7.44	7.53	7.51	
Carbon	82.95	82.95	83.73	
Hydrogen	5.36	5.53	5.55	

Authorising Signature:

**Date completed**	28.11.22
**Signature**	[illegible]
**comments**	

Figure **S5.** Elemental analysis of **MeTPA-BQ**

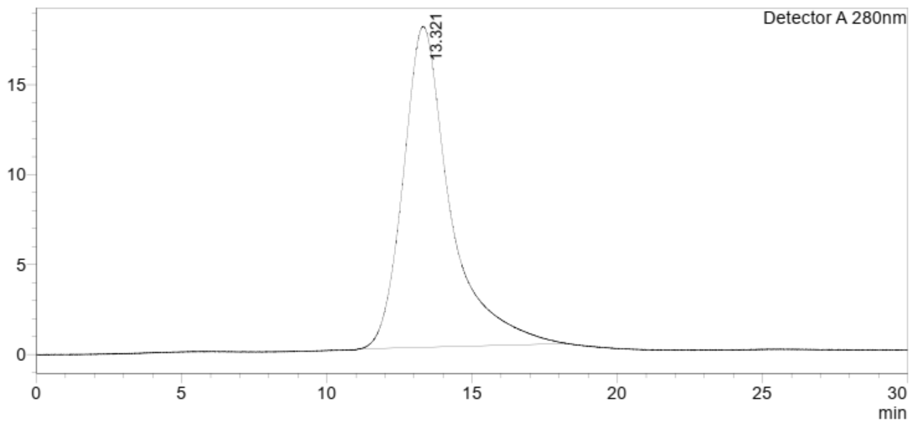

<Peak Table>

Detector A 280nm

Peak#	Ret. Time	Area	Height	Area%	Area/Height	Width at 5% Height
1	13.321	2010840	17839	100.000	112.719	4.791
Total		2010840	17839	100.000		

Figure **S6**. HPLC trace of **MeTPA-BQ**

1,2-bis(4-(bis(4-(tert-butyl)phenyl)amino)phenyl)ethane-1,2-dione (4)

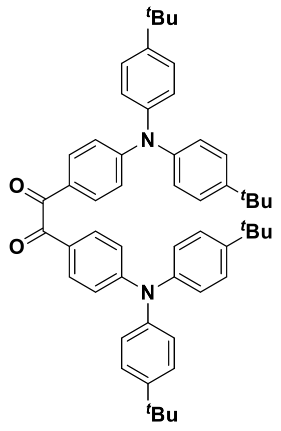

A mixture of 1,2-bis(4-bromophenyl)ethane-1,2-dione (1.00 g, 2.72 mmol, 1.0 equiv.), bis(4-(*tert*-butyl)phenyl)amine (1.61 g, 5.71 mmol, 2.1 equiv.), K_2CO_3 (1.50 g, 10.87 mmol,2.2 equiv.), $Pd(OAc)_2$ (73. 2 mg, 0.33 mmol, 0.12 equiv.) and tri-*tert*-butylphosphonium tetrafluoroborate (284 mg, 0.98 mmol, 0.36 equiv.) were placed in a dried Schlenk flask and purged with N_2. Toluene (30 mL) was added to the mixture under an N_2 atmosphere. The mixture was stirred at

100 °C for 12 h, then cooled to room temperature and quenched with brine (80 mL). The product was extracted with CH_2Cl_2, washed several times with water (2 × 100 mL), dried over anhydrous Na_2SO_4 and concentrated *in vacuo*. The crude mixture was purified by silica gel flash column chromatography using DCM : hexane = 2:1 as the eluent to afford the desired compound as a white solid. **Yield**: 74%. **R_f:** 0.33 (DCM : hexane = 2:1 on silica gel). **Mp**: 252-254 °C. **^{1}H NMR (400 MHz, $CDCl_3$) δ (ppm)**: 7.76 (d, *J* = 9.0 Hz, 4H), 7.38 – 7.33 (m, 8H), 7.14 – 7.08 (m, 8H), 6.92 (d, *J* = 9.1 Hz, 4H), 1.34 (s, 36H). **^{13}C NMR (101 MHz, $CDCl_3$) δ (ppm)**: 193.60, 153.69, 148.30, 143.09, 131.60, 126.56, 126.01, 124.70, 118.06, 77.35, 77.03, 76.72, 34.51, 31.39. **HR-MS[M+H]$^+$ Calculated: (**$C_{55}H_{60}N_2O_2$) 769.4728; **Found**: 769.4728.

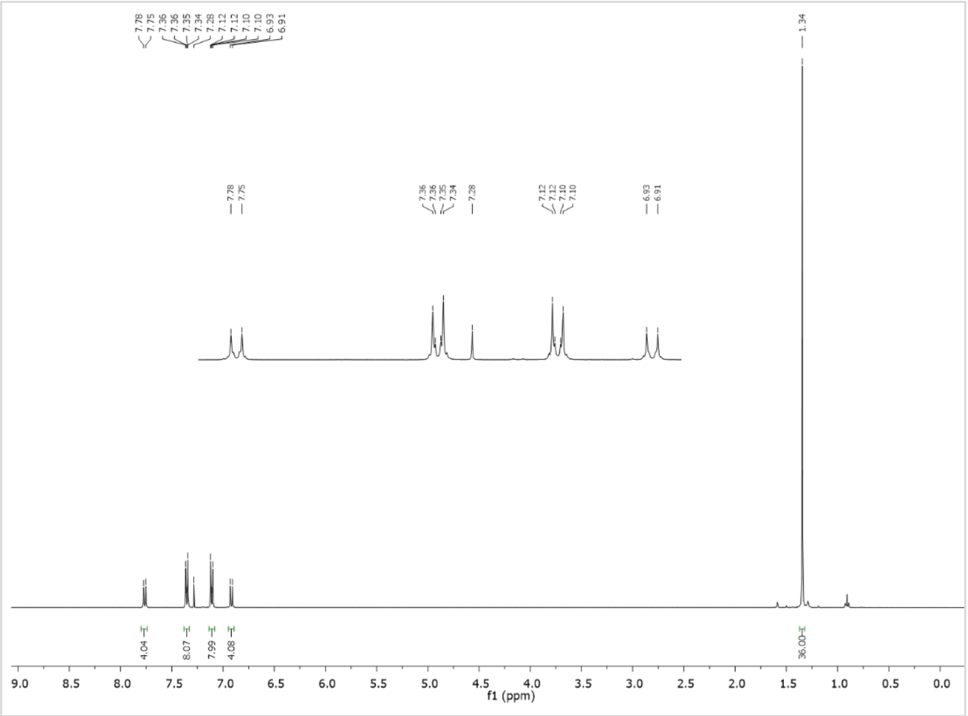

Figure **S7**. ^{1}H NMR of **4** in $CDCl_3$

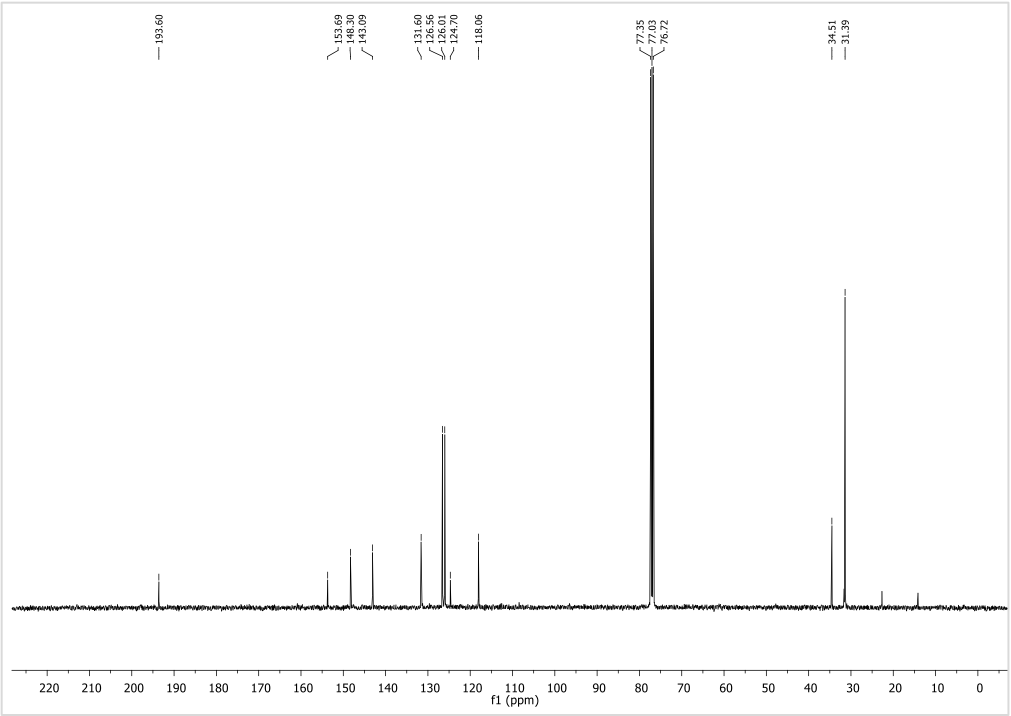

Figure **S8**. ^{13}C NMR of **4** in $CDCl_3$

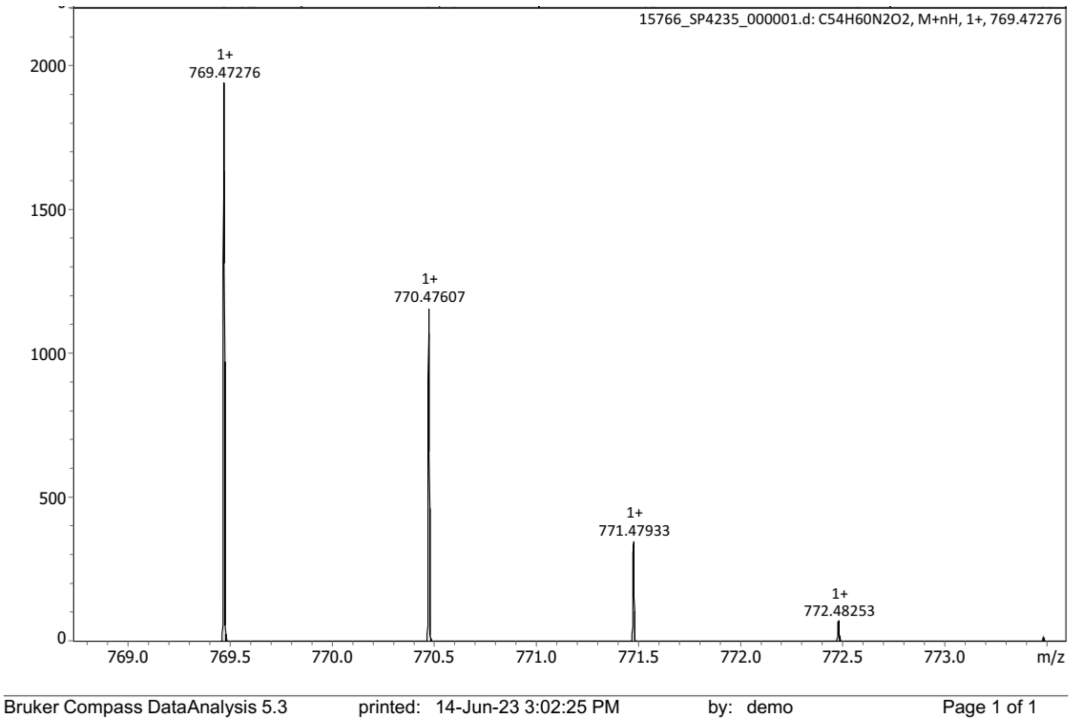

Figure **S9**. HRMS of **4**

2,3-bis(4-(bis(4-(*tert*-butyl)phenyl)amino)phenyl)benzo[g]quinoxaline-5,10-dione (tBuTPA-BQ)

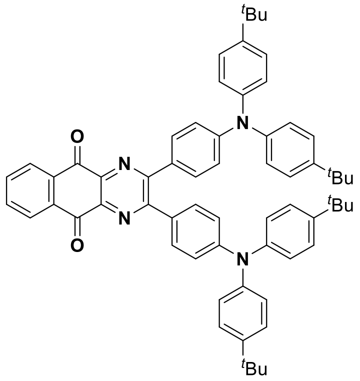

The compound **tBuTPA-BQ** was synthesized by an analogous procedure to that used for the synthesis of **MeTPA-BQ**. The stoichiometry of starting materials was 2,3-diamino-1,4-naphthoquinone (**2**) (100 mg; 531 μmol, 1 equiv.) and 1,2-bis(4-(bis(4-(*tert*-butyl)phenyl)amino)phenyl)ethane-1,2-dione (**4**) (500 mg, 584 μmol, 1.1 equiv.) and glacial acetic acid (5 mL). The crude mixture was purified by silica gel flash column chromatography using DCM : hexane = 3:1 as eluent to afford the desired compound as a red solid. **Yield:** 89%. **R_f:** 0.3 (DCM : hexane = 2:1 on silica gel). **Mp:** 342-344 °C. **^{1}H NMR (400 MHz, $CDCl_3$) δ (ppm):** 8.43 (dd, *J* = 5.7, 3.3 Hz, 2H), 7.88 (dd, *J* = 5.8, 3.3 Hz, 2H), 7.63 (d, *J* = 8.7 Hz, 4H), 7.37 – 7.30 (m, 8H), 7.10 (d, *J* = 8.6 Hz, 8H), 6.99 (d, *J* = 8.7 Hz, 4H), 1.36 (s, 36H). **^{13}C NMR (125 MHz, $CDCl_3$) δ (ppm):** 181.58, 156.15, 150.06, 147.03, 144.03, 141.17, 134.59, 133.31, 130.94, 129.22, 129.06, 128.25, 127.61, 126.25, 125.22, 120.05, 77.36, 77.04, 76.72, 34.43, 31.45. **HR-MS$[M+H]^+$ Calculated:** ($C_{64}H_{64}N_4O_2$) 920.5029; **Found:** 920.50809. **Anal**. Calcd. for $C_{64}H_{64}N_4O_2$: C, 83.44%; H, 7.00%; N, 6.08%. Found: C, 83.92%; H, 6.98%; N, 6.01%. **HPLC-GPC** purity: 100%. Retention time: 13.15 min.

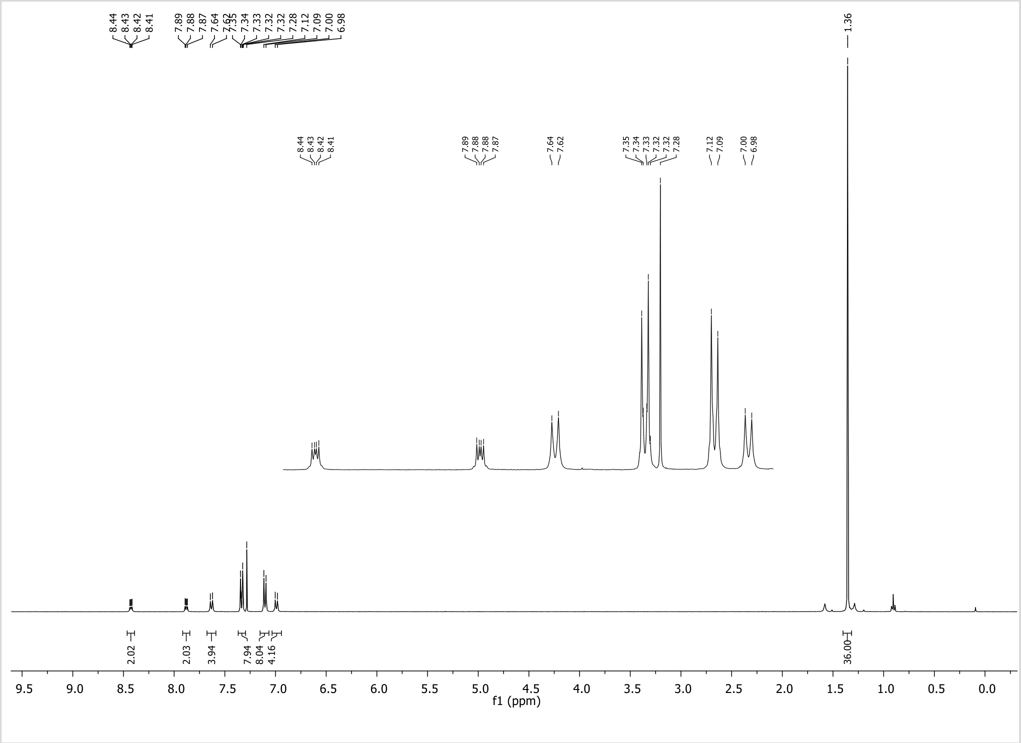

Figure **S10**. ^{1}H NMR of **tBuTPA-BQ** in $CDCl_3$

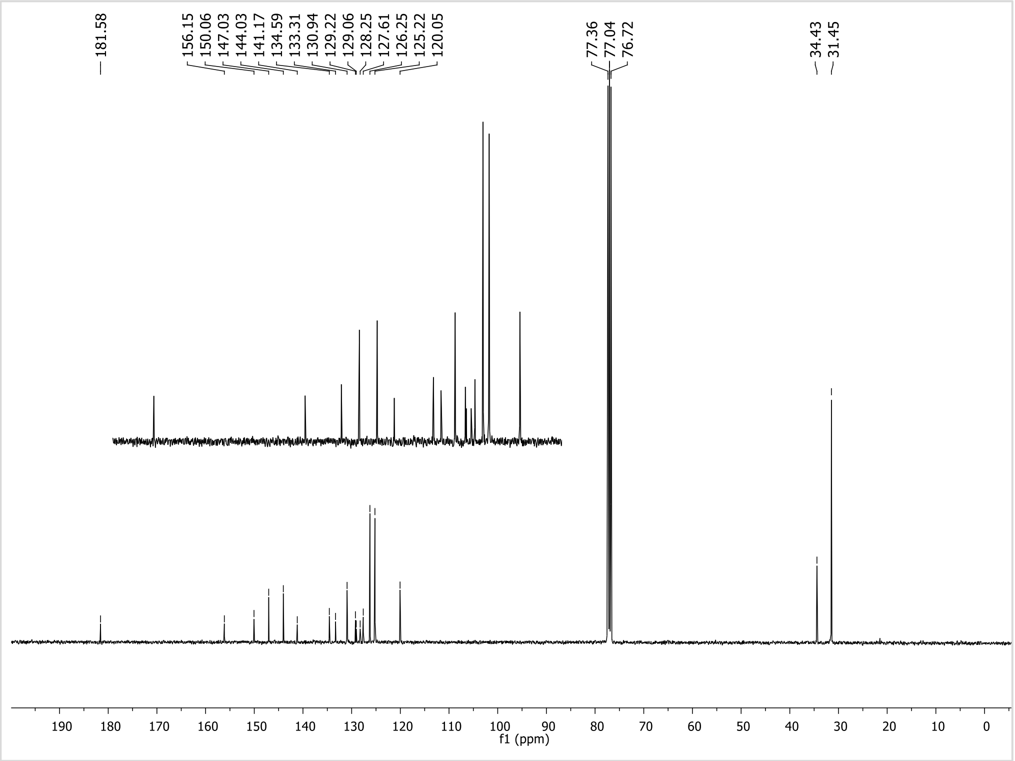

Figure **S11**. ^{13}C NMR of **tBuTPA-BQ** in $CDCl_3$

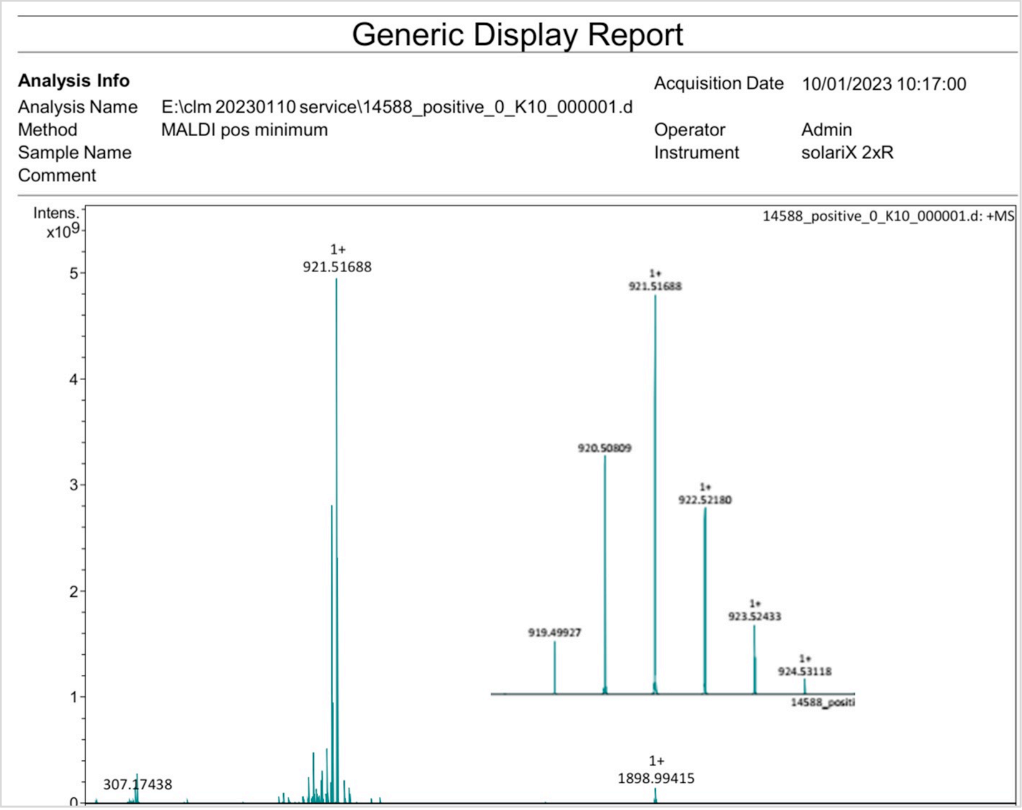

Figure **S12**. HRMS of **tBuTPA-BQ** (inset shows isotopic distribution pattern of molecular ion)

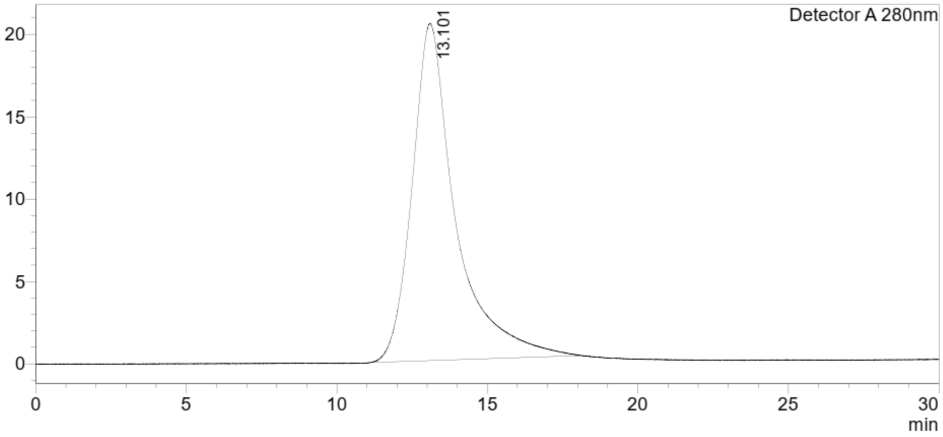

<Peak Table>

Detector A 280nm

Peak#	Ret. Time	Area	Height	Area%	Area/Height	Width at 5% Height
1	13.101	2049070	20456	100.000	100.169	4.418
Total		2049070	20456	100.000		

Figure **S13**. HPLC trace of **tBuTPA-BQ**

Single ☐ **Duplicate** ■ **Triplicate** ☐

Analysis Result:

Element	Expected %	Found (1)	Found (2)	Found (3)
Nitrogen	6.08	6.01	6.02	
Carbon	83.44	83.85	84.05	
Hydrogen	7.00	6.96	7.00	

Authorising Signature:

Date completed	09-05-23
Signature	[illegible]
comments	

Figure **S14.** Elemental analysis of **tBuTPA-BQ**

2,3-bis(4'-(diphenylamino)-[1,1'-biphenyl]-4-yl)benzo[g]quinoxaline-5,10-dione (TPPA-BQ)

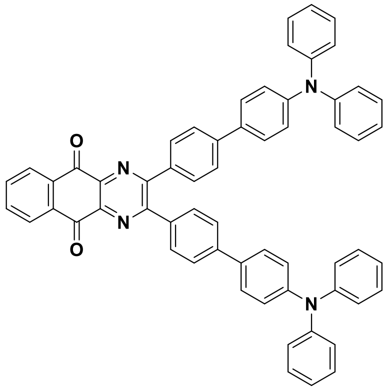

The compound **TPPA-BQ** was synthesized by an analogous procedure to that used for the synthesis of **MeTPA-BQ**. The stoichiometry of starting materials was 2,3-diamino-1,4-naphthoquinone (**2**) (200 mg, 1.06 mmol, 1 equiv.) and 1,2-bis(4'-(diphenylamino)-[1,1'-biphenyl]-4-yl)ethane-1,2-dione (**5**) (814 mg, 1.17 mmol, 1.1 equiv.) and glacial acetic acid (10 mL). The crude mixture was purified by silica gel flash column chromatography using DCM:hexane = 3:1 as eluent to afford the desired compound as a white solid. **Yield:** 82%. **R_f:** 0.22 (DCM : hexane = 2:1 on silica gel). **Mp:** 197-200 °C. **^{1}H NMR (400 MHz, $CDCl_3$) δ (ppm):**

8.45 (dd, J = 5.8, 3.3 Hz, 1H), 7.90 (dd, J = 5.8, 3.3 Hz, 1H), 7.79 (d, J = 8.5 Hz, 2H), 7.60 (d, J = 8.6 Hz, 2H), 7.51 (d, J = 8.8 Hz, 2H), 7.31 – 7.24 (m, 4H), 7.16 – 7.11 (m, 5H), 7.05 (t, J = 7.3 Hz, 2H). **^{13}C NMR (125 MHz, $CDCl_3$) δ (ppm):** 181.34, 156.57, 147.90, 147.49, 142.32, 141.93, 135.48, 134.88, 133.40, 133.28, 130.69, 129.37, 127.82, 127.76, 126.58, 124.64, 123.59, 123.22, 77.39, 77.07, 76.75. **HR-MS[M+Na]$^+$ Calculated:** ($C_{60}H_{40}N_4O_2$) 848.3146; **Found:** 848.3121. **Anal**. Calcd. for $C_{60}H_{40}N_4O_2$: C, 84.88%; H, 4.75%; N, 6.60%. **Found:** C, 84.69%; H, 4.73%; N, 6.25%. **HPLC:** 100%, retention time: 4.84 minutes in 98% MeCN/2% H_2O.

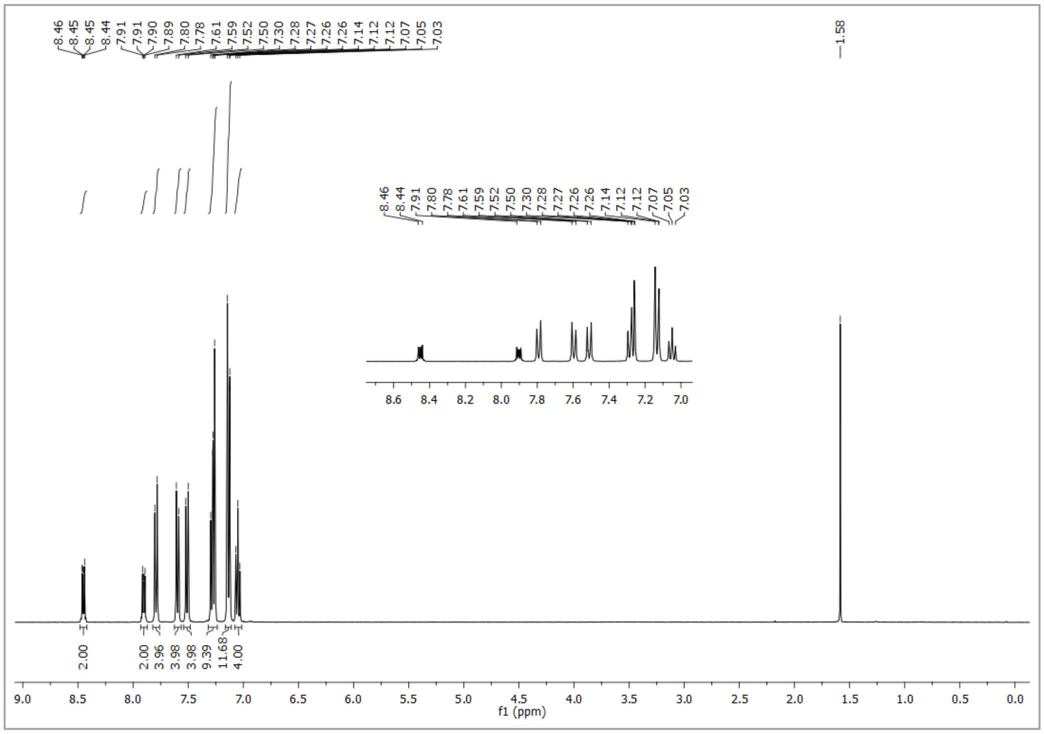

Figure S15. ^{1}H NMR of **TPPA-BQ** in $CDCl_3$.

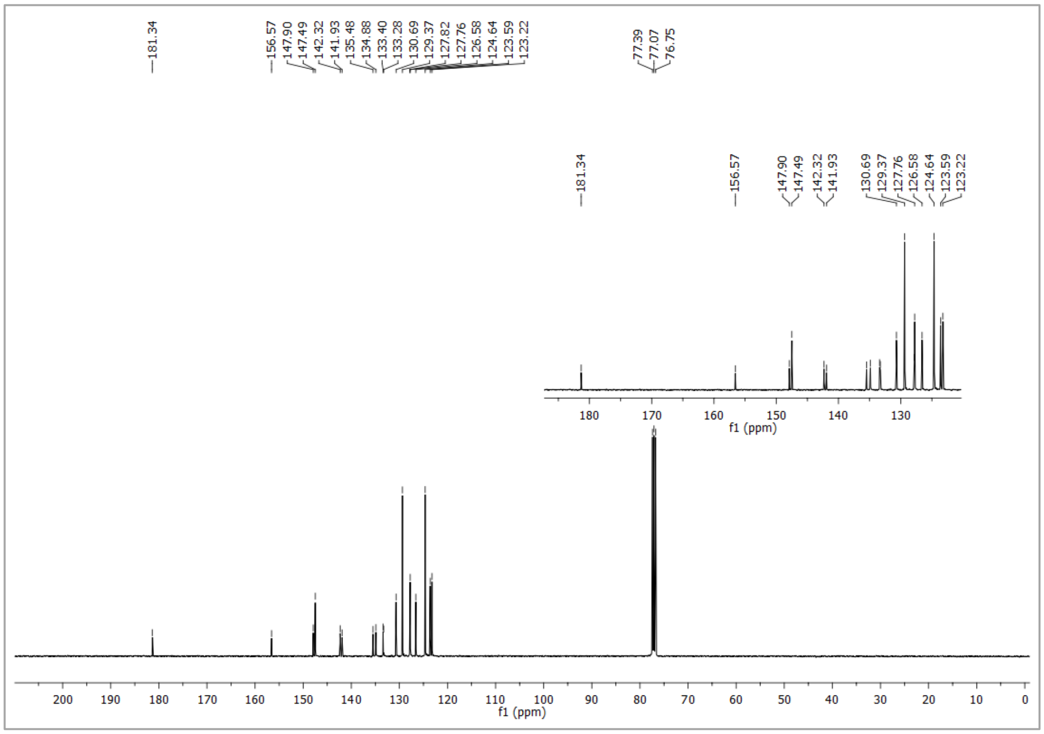

Figure S16. ^{13}C NMR of **TPPA-BQ** in $CDCl_3$.

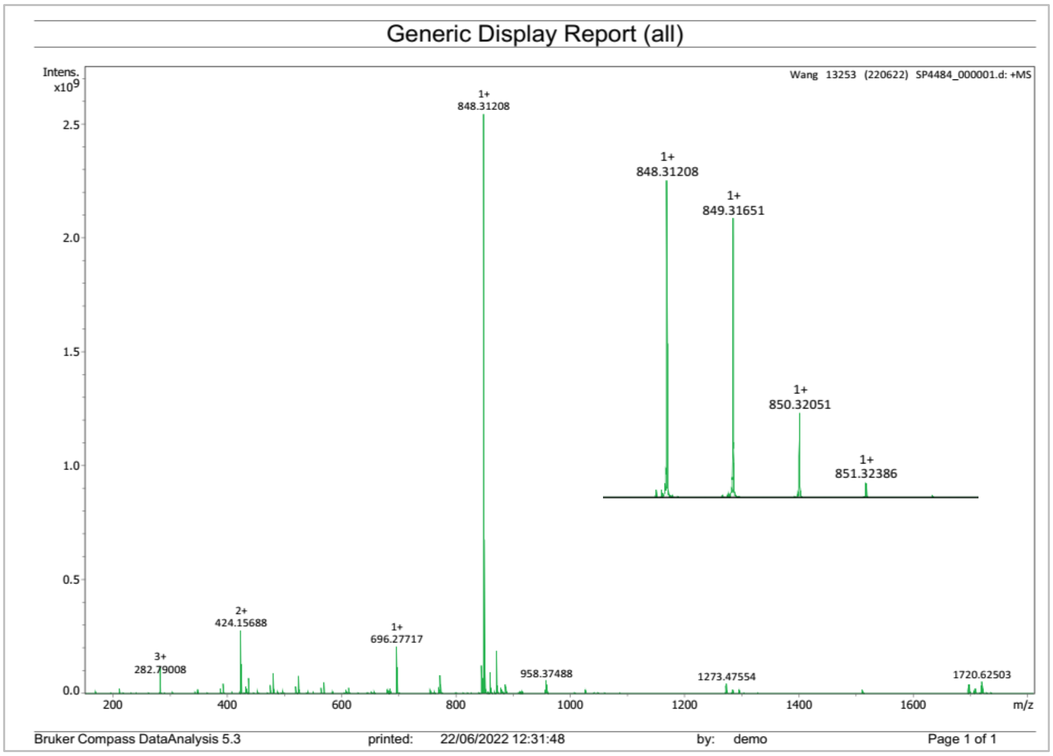

Figure S17. HRMS of **TPPA-BQ** (inset shows the isotopic distribution pattern of molecular ion)

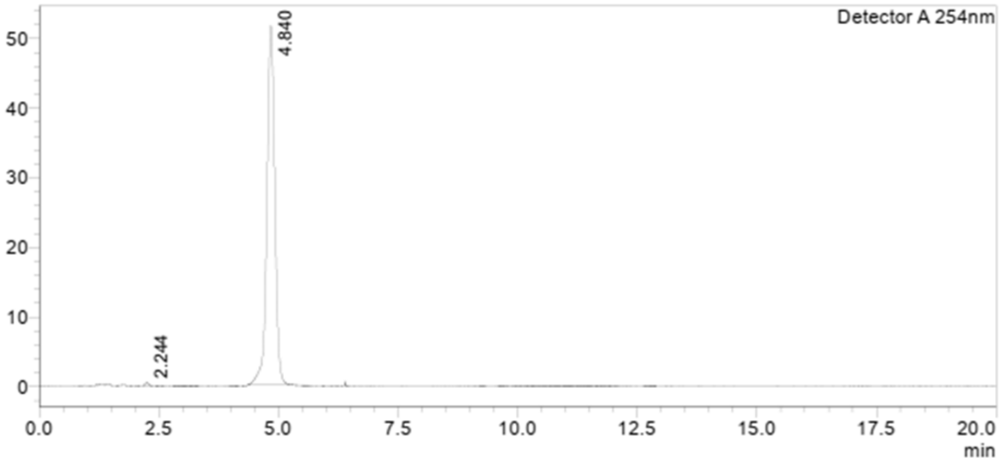

<Peak Table>

Detector A 254nm

Peak#	Ret. Time	Area	Height	Area%	Area/Height	Width at 5% Height
1	2.244	2037	379	0.341	5.368	0.168
2	4.840	594369	51484	99.659	11.545	0.412
Total		596406	51863	100.000		

Figure **S18**. HPLC trace of **TPPA-BQ**

Analysis type:

Single ☐ **Duplicate** ■ **Triplicate** ☐

Analysis Result:

Element	Expected %	Found (1)	Found (2)	Found (3)
Nitrogen	6.60	6.34	6.17	
Carbon	84.88	84.54	84.85	
Hydrogen	4.75	4.74	4.72	

Authorising Signature:

**Date completed**	30.09.22
**Signature**	[illegible]
**comments**	

Figure **S19**. Elemental analysis of **TPPA-BQ**

X-ray crystallography

X-ray diffraction data for **MeTPA-BQ** and **tBuTPA-BQ** were collected at 125 K using a Rigaku MM-007HF High Brilliance RA generator/confocal optics [Cu Kα radiation (λ = 1.54187 Å)] with XtaLAB P200 diffractometer. Intensity data were collected using ω steps accumulating area detector images spanning at least a hemisphere of reciprocal space. Data for both compounds were collected using CrystalClear[13] and processed (including correction for Lorentz, polarization and absorption) using CrysAlisPro.[14] Structures were solved by direct methods (SIR2011[15]) and refined by full-matrix least-squares against F^2 (SHELXL-2019/3[16]). Non-hydrogen atoms were refined anisotropically, and hydrogen atoms were refined using a riding model. Two of the *t*-butyl groups in **tBuTPA-BQ** showed rotational disorder, and the methyl sites were split over two locations, the minor component of one disordered *t*-butyl group requiring isotropic refinement. The disordered atoms were refined with restraints to bond distances and thermal motion. All calculations were performed using the Olex2[17] interface. Selected crystallographic data are presented in Table **S1**. CCDC 2296442-2296443 contains the supplementary crystallographic data for this paper. These data can be obtained free of charge from The Cambridge Crystallographic Data Centre via www.ccdc.cam.ac.uk/structures.

Table **S1**. Selected crystallographic data.

	**MeTPA-BQ**	**tBuTPA-BQ**
formula	$C_{52}H_{40}N_4O_2$	$C_{64}H_{64}N_4O_2$
fw	752.88	921.19
crystal description	Red needle	Orange plate
crystal size [mm^3]	0.24×0.02×0.01	0.10×0.03×0.01
space group	$P\bar{1}$	$P\bar{1}$
a [Å]	10.0726(3)	5.9658(2)
b [Å]	14.3581(4)	17.8685(7)
c [Å]	15.3628(4)	25.1599(10)
α [°]	109.639(3)	76.932(4)
β [°]	95.666(2)	86.233(3)
γ [°]	108.274(3)	86.248(3)
vol [Å]^3	1934.60(11)	2603.41(19)
Z	2	2
ρ (calc) [g/cm^3]	1.292	1.175
μ [mm^{-1}]	0.620	0.545
F(000)	792	984
reflections collected	23164	30125
independent reflections (R_{int})	7654 (0.0307)	10255 (0.0948)
parameters, restraints	527, 0	687, 207
GoF on F^2	1.051	1.020
R_1 [$I > 2\sigma(I)$]	0.0416	0.0604
wR_2 (all data)	0.1179	0.1678
largest diff. peak/hole [e/Å^3]	0.209, -0.232	0.310, -0.296

Table **S2**. Particle (blue) and hole (red) for S_1, T_1 and T_2 states estimated from S_0 equilibrium geometries.

Compound	**S_1**	**T_1**
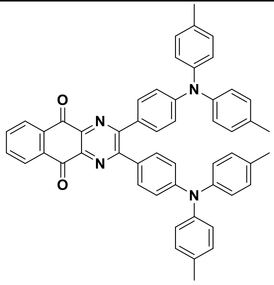	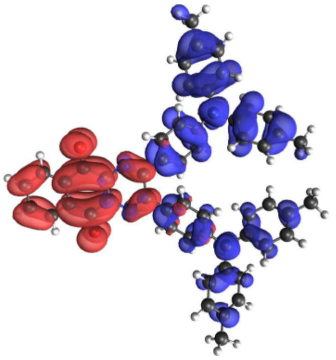	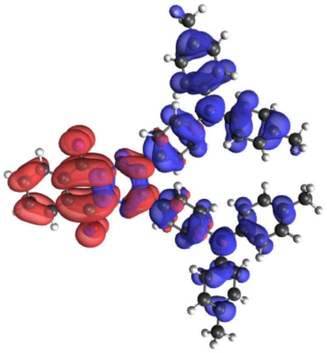
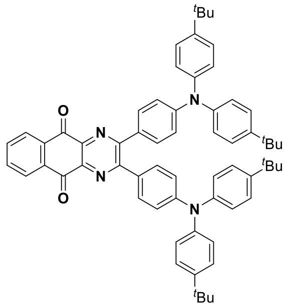	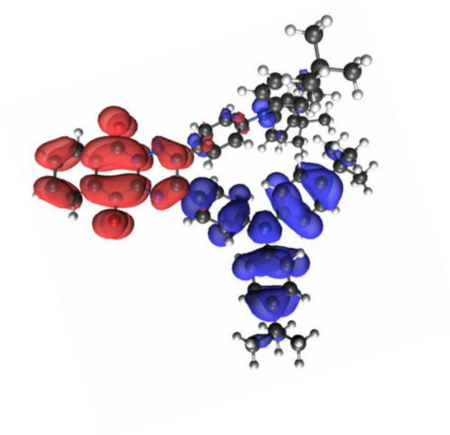	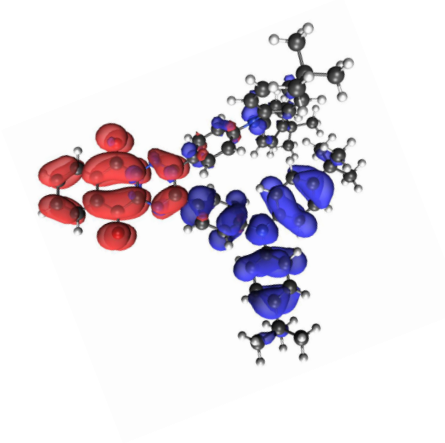
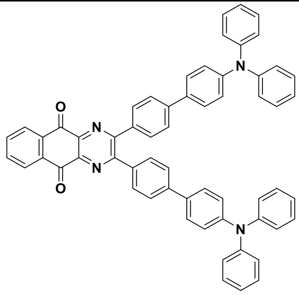	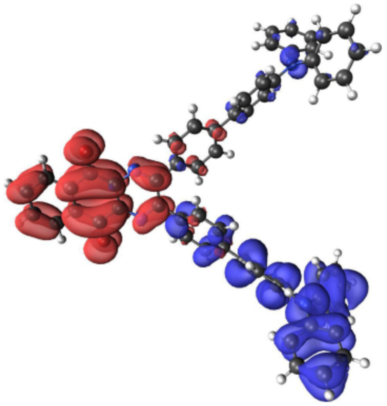	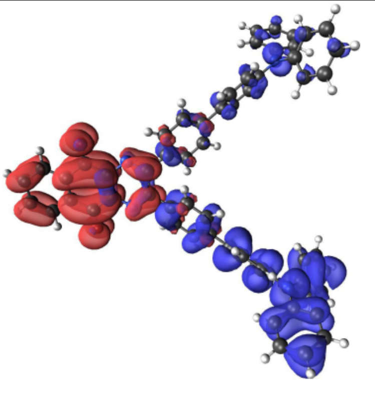

Table **S3**. Electrochemical data of **MeTPA-BQ**, **tBuTPA-BQ** and **TPPA-BQ** compounds.

Emitter	E^{ox} / V[a]	E^{red} / V[a]	HOMO / eV[b]	LUMO / eV[b]	ΔE_{HL} / eV[c]
**MeTPA-BQ**	0.94	-0.78	-5.28	-3.56	1.72
**tBuTPA-BQ**	0.91	-0.79	-5.25	-3.55	1.70
**TPPA-BQ**	0.97	-0.76	-5.31	-3.58	1.73

[a]Obtained from DPV peaks and referenced with respect to SCE (Fc/Fc^+ = 0.46 V for DCM).[18]
[b]$E_{HOMO/LUMO}$ = -($E^{ox/red}$(vs Fc/Fc^+) + 4.8) eV.[19] [c]$\Delta E_{HL} = |E_{HOMO}-E_{LUMO}|$.

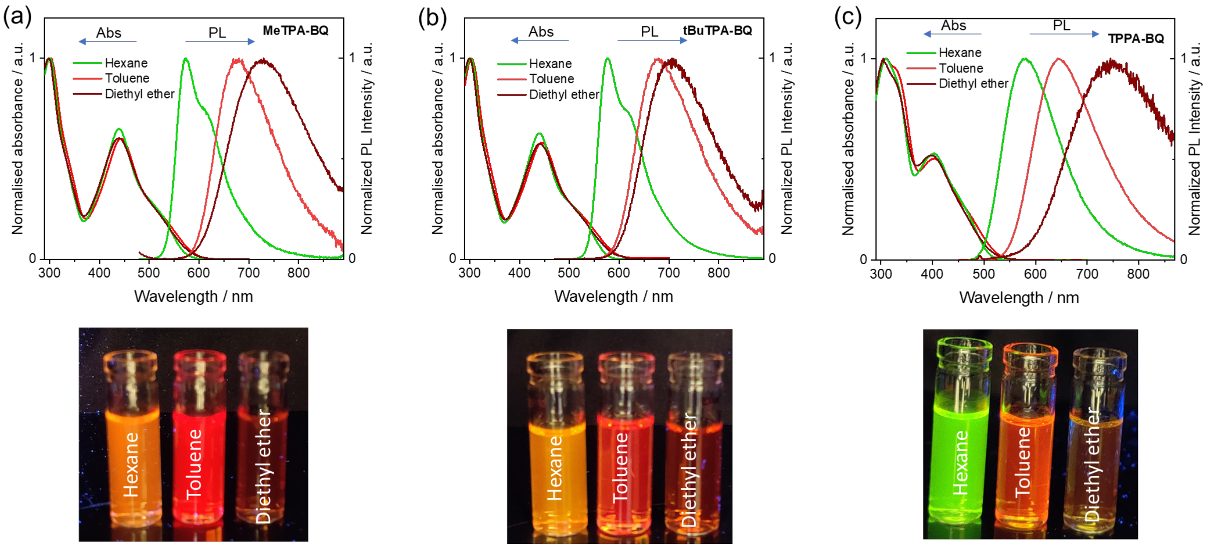

Figure **S20**. Absorption and PL spectra of **MeTPA-BQ**, **tBuTPA-BQ** and **TPPA-BQ** in different solvents of polarity (λ_{exc} = 450 nm).

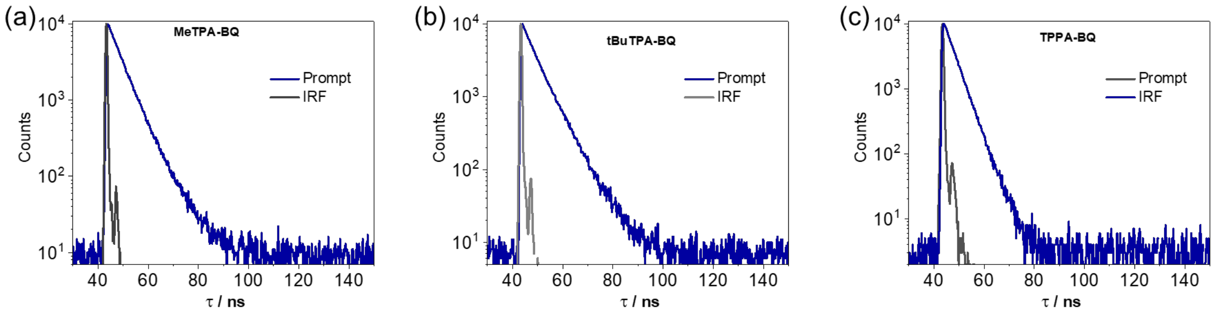

Figure **S21.** TRPL of (a) **MeTPA-BQ**, (b) **tBuTPA-BQ** and (c) **TPPA-BQ** (λ_{exc} = 375 nm) in toluene.

Table **S4**. Photoluminescence quantum yield (Φ_{PL}) of the **MeTPA-BQ**, **tBuTPA-BQ** and **TPPA-BQ** in doped CBP films.

	2 wt%	3 wt%	5 wt%	10 wt%
	Φ_{PL} (N_2 / O_2) / (%)	Φ_{PL} (N_2 / O_2) / (%)	Φ_{PL} (N_2 / O_2) / (%)	Φ_{PL} (N_2 / O_2) / (%)
**MeTPA-BQ**	42 / 32	31 / 22	19 / 12	13 / 9
**tBuTPA-BQ**	41 / 30	34 / 26	23 / 16	15 / 11
**TPPA-BQ**	39 / 28	30 / 23	21 / 11	10 / 8

Φ_{PL} was recorded under air/ N_2 atmosphere using an integrating sphere for 2 wt% doped films in CBP (λ_{exc} = 340 nm).

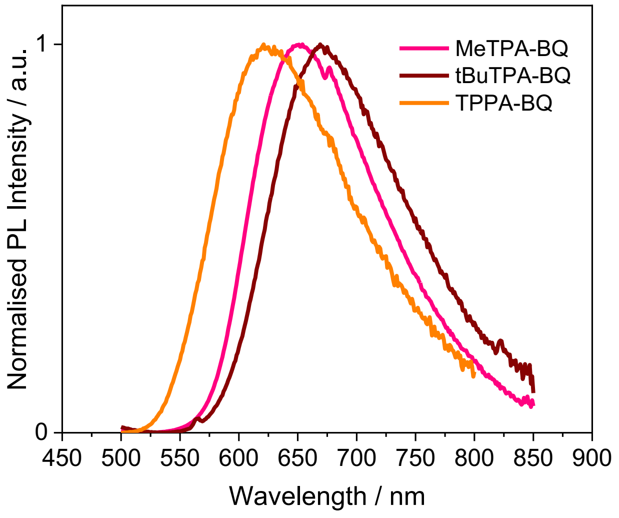

Figure **S22**. PL spectra of **MeTPA-BQ**, **tBuTPA-BQ** and **TPPA-BQ** in 2 wt% doped films (λ_{exc} = 340 nm).

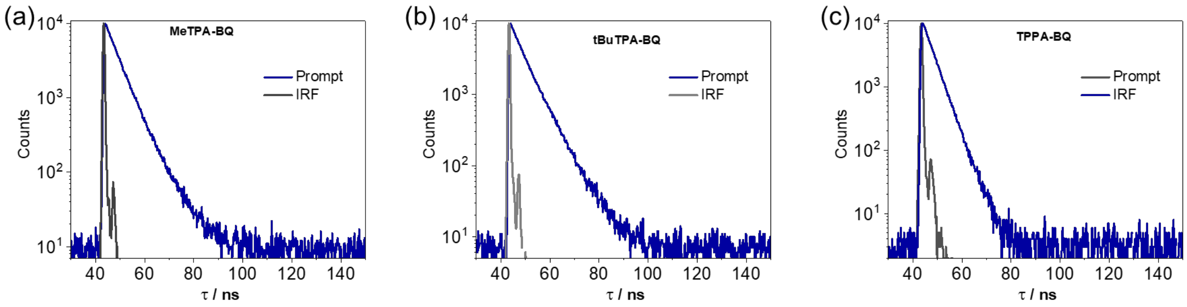

Figure **S22.** TRPL of (a) **MeTPA-BQ**, (b) **tBuTPA-BQ** and (c) **TPPA-BQ** (λ_{exc} = 375 nm).

Table **S5**. Decay components of prompt emission and delayed emission and average lifetimes of of **MeTPA-BQ**, **tBuTPA-BQ** and **TPPA-BQ** in doped CBP films.

	τ_p (relative contribution) / ns [a]	$\tau_{av,\,p}$ / ns [b]	χ^2	τ_d (relative contribution) / µs [c]	$\tau_{av,\,d}$ / µs [d]	χ^2
**MeTPA-BQ**	τ_1 = 4.2 (22%)<br>τ_2 = 9.1 (78%)	8.0	0.99	τ_2 = 5.9 (46%)<br>τ_3 = 69.2 (54%)	40.1	1.3
**tBuTPA-BQ**	τ_1 = 3.5 (22%)<br>τ_2 = 11.6 (78%)	9.9	1.05	τ_1 = 13.9 (14%)<br>τ_2 = 165.0 (86%)	143.8	1.2
**TPPA-BQ**	τ_1 = 8.0 (52%)<br>τ_2 = 16.7 (48%)	12.2	0.98	τ_1 = 8.3 (37%)<br>τ_2 = 63.3 (63%)	42.8	1.1

[a]Prompt average lifetime (τ_p) was recorded using time-correlated single photon counting (TCSPC) (λ_{exc} = 375 nm), [c]Delayed average lifetime (τ_d) was recorded using a microsecond flash lamp (λ_{exc} = 340 nm). [b,d] Average lifetime calculated using $\Sigma(A_n\tau_n^2)/\Sigma(A_n\tau_n)$, where A_n is the coefficient of the exponential functions.

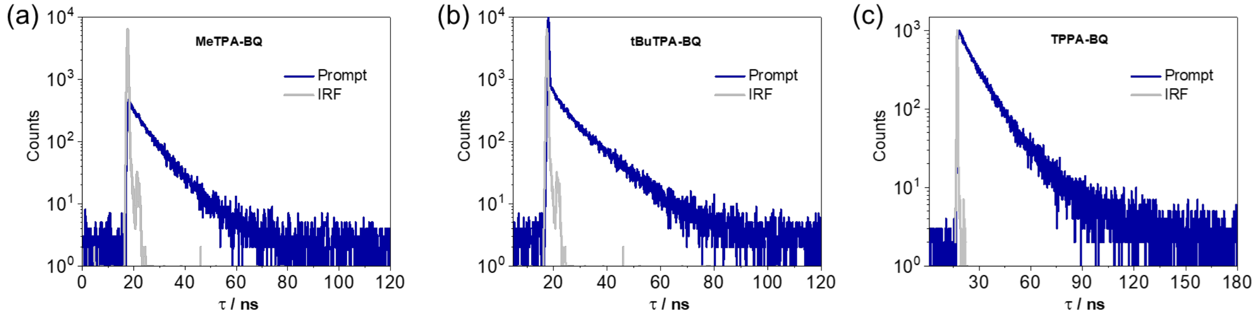

Figure **S23.** TRPL of (a) **MeTPA-BQ**, (b) **tBuTPA-BQ** and (c) **TPPA-BQ** (λ_{exc} = 375 nm) in 2 wt% doped CBP films.

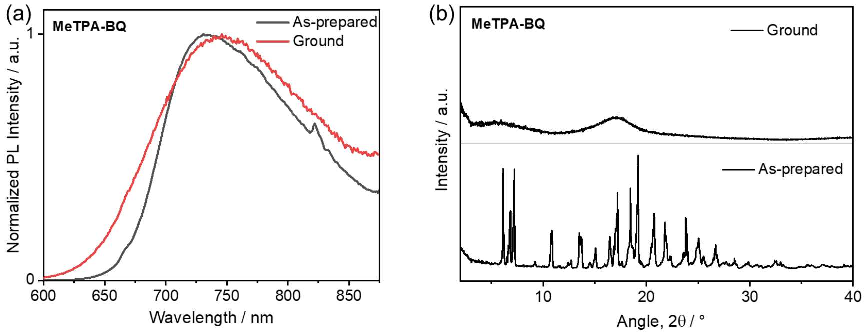

Figure **S24**. (a) PL of as prepared and ground of **MeTPA-BQ** (λ_{exc} = 450 nm) and their corresponding (b) PXRD spectra.

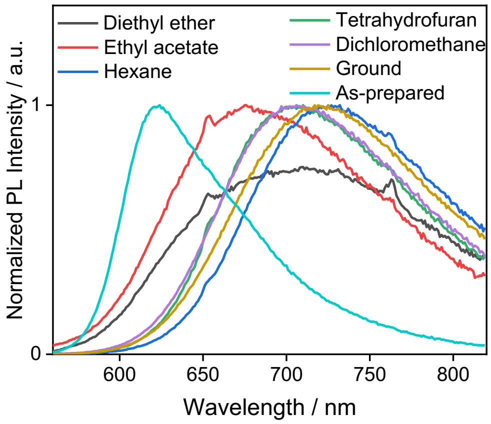

Figure **S25**. PL spectra of ground **tBuTPA-BQ** upon exposure to different solvents (λ_{exc} = 450 nm).

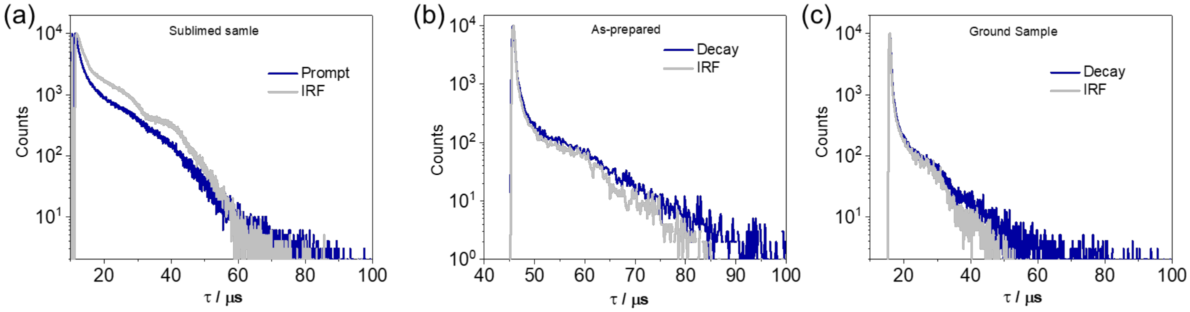

Figure **S26**. TRPL spectra of (a) sublimed (b) as-prepared and (c) ground samples of **tBuTPA-BQ** (indicating no PL decay observed for these samples).

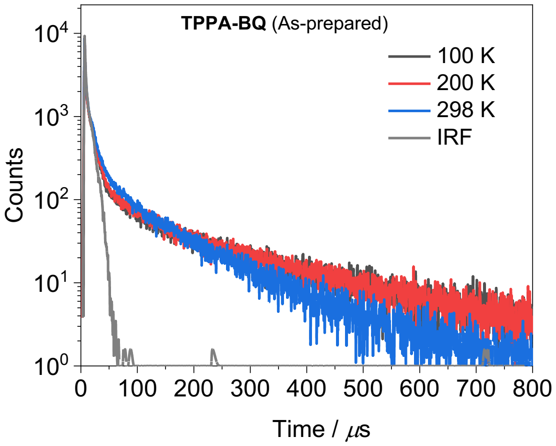

Figure **S27**. TRPL spectra of **TPPA-BQ** (as-prepared sample) (λ_{exc} = 450 nm).

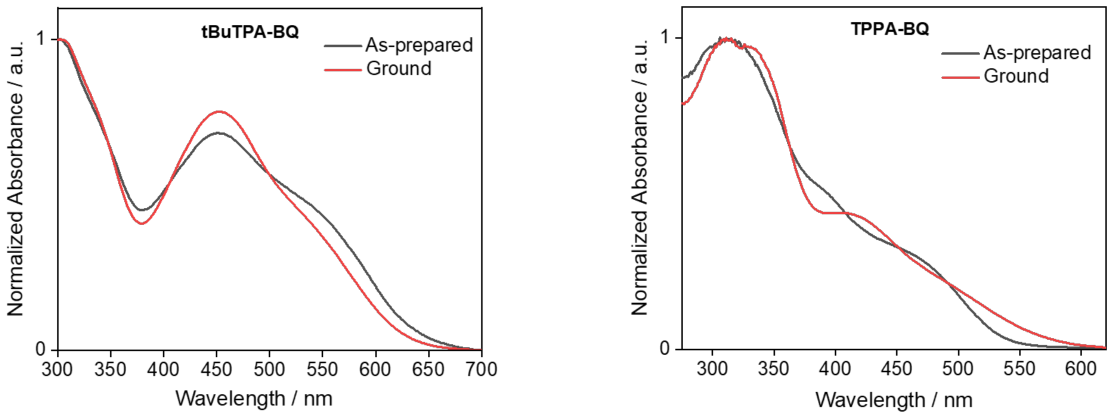

Figure **S28**. Absorption spectra of the as prepared and ground samples of the **tBuTPA-BQ** and **TPPA-BQ**.

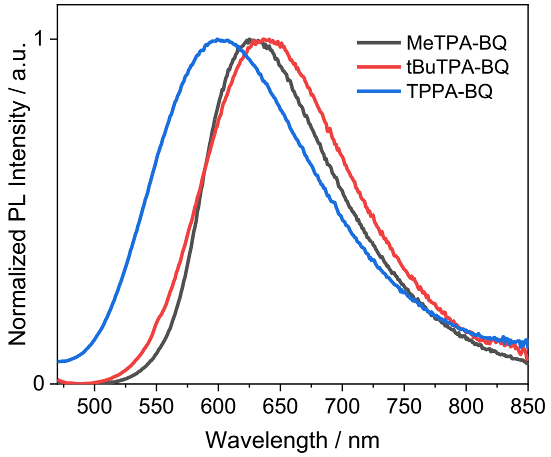

Figure **S29**. PL spectra of **MeTPA-BQ**, **tBuTPA-BQ** and **TPPA-BQ** in 0.5 wt% doped films in PMMA (λ_{exc} = 450 nm).

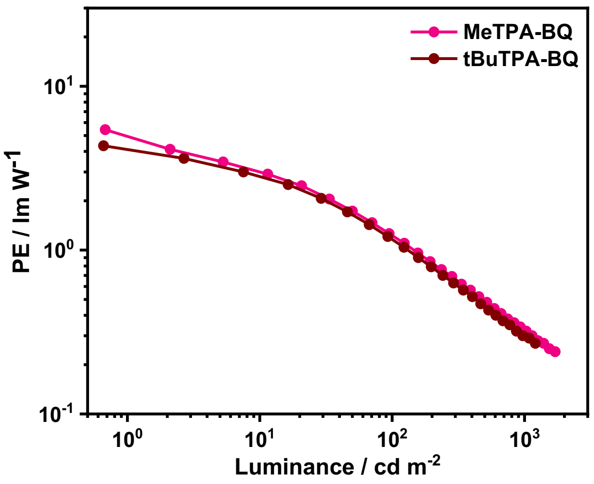

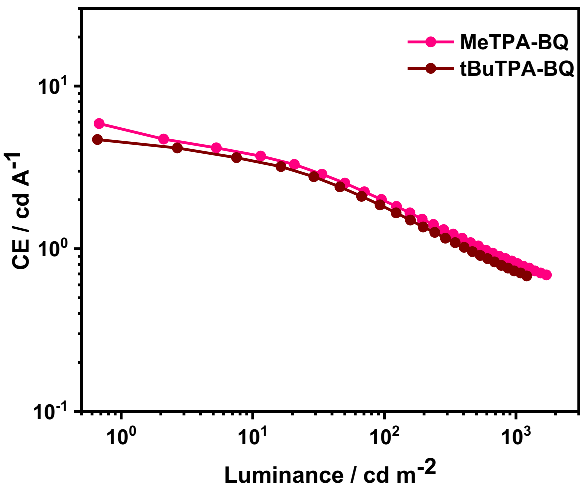

Figure **S30**. a) Power efficiency versus luminance and b) current efficiency versus luminance curves for devices **MeTPA-BQ** (2 wt%) and **tBuTPA-BQ** (2 wt%) doped in CBP host.

Table **S6.** EL performance of representative deep-red TADF OLEDs with emission peak between 650 and 670 nm.

Emitter	EQE_{max}	CIE (x, y)	λ_{max}	Reference
DDTPACz-DCPP	13.1	(0.63, 0.37)	652	[20]
*p*TPA-DPPZ	12.3	(0.67, 0.33)	652	[21]
PXZ-10-DPPZ	0.8	(0.65, 0.35)	655	[22]
TPA-QCN	12.8	(0.64, 0.35)	656	[23]
BPPZ-PXZ	2.5	(0.65, 0.35)	656	[24]
PIPAQ	2.1	(0.64, 0.36)	656	[25]
TPAAP	14.1	-	657	[26]
BTZ-DMAC	5.3	(0.63, 0.36)	658	[27]
*p*CNQ-TPA	30.3	(0.69, 0.31	660	[28]
TPA-PPDCN	20.2	(0.68, 0.32)	664	[29]
PA-DCPP	9.8	(0.68, 0.32)	668	[30]
APDC-DTPA	12.3	-	668	[31]
Da-CNBPz	15.0	(0.66, 0.34)	670	[32]
**MeTPA-BQ**	**10.1**	**(0.64, 0.34)**	**650**	**Present work**
**tBuTPA-BQ**	**8.5**	**(0.66, 0.34)**	**670**	**Present work**

References

1. Y. Wang, Z. He, G. Chen, T. Shan, W. Yuan, P. Lu and Y. Zhang, D-A structured high efficiency solid luminogens with tunable emissions: Molecular design and photophysical properties, *Chin. Chem. Lett.*, 2017, **28**, 2133-2138.
2. Q. Fan, J. Cui, Y. Liu, W. Su, Y. Wang, H. Tan, D. Yu, H. Gao, X. Deng and W. Zhu, Synthesis and photovoltaic properties of two star-shaped molecules involving phenylquinoxaline as core and triphenylamine and thiophene units as arms, *Synth. Met.*, 2015, **204**, 25-31.
3. B. Chesneau, M. Hardouin-Lerouge and P. Hudhomme, A Fused Donor−Acceptor System Based on an Extended Tetrathiafulvalene and a Ruthenium Complex of Dipyridoquinoxaline, *Org. Lett.*, 2010, **12**, 4868-4871.
4. M. J. Frisch, G. W. Trucks, H. B. Schlegel, G. E. Scuseria, M. A. Robb, J. R. Cheeseman, G. Scalmani, V. Barone, G. A. Petersson, H. Nakatsuji, X. Li, M. Caricato, A. V. Marenich, J. Bloino, B. G. Janesko, R. Gomperts, B. Mennucci, H. P. Hratchian, J. V. Ortiz, A. F. Izmaylov, J. L. Sonnenberg, Williams, F. Ding, F. Lipparini, F. Egidi, J. Goings, B. Peng, A. Petrone, T. Henderson, D. Ranasinghe, V. G. Zakrzewski, J. Gao, N. Rega, G. Zheng, W. Liang, M. Hada, M. Ehara, K. Toyota, R. Fukuda, J. Hasegawa, M. Ishida, T. Nakajima, Y. Honda, O. Kitao, H. Nakai, T. Vreven, K. Throssell, J. A. Montgomery Jr., J. E. Peralta, F. Ogliaro, M. J. Bearpark, J. J. Heyd, E. N. Brothers, K. N. Kudin, V. N. Staroverov, T. A. Keith, R. Kobayashi, J. Normand, K. Raghavachari, A. P. Rendell, J. C. Burant, S. S. Iyengar, J. Tomasi, M. Cossi, J. M. Millam, M. Klene, C. Adamo, R. Cammi, J. W. Ochterski, R. L. Martin, K. Morokuma, O. Farkas, J. B. Foresman and D. J. Fox, Gaussian 16 Rev. C.01.*Journal*, 2016.
5. C. Adamo and V. Barone, Toward reliable density functional methods without adjustable parameters: The PBE0 model, *J. Chem. Phys.*, 1999, **110**, 6158-6170.
6. G. A. Petersson, T. G. Tensfeldt and J. A. Montgomery, Jr., A complete basis set model chemistry. III. The complete basis set-quadratic configuration interaction family of methods, *J. Chem. Phys.*, 1991, **94**, 6091-6101.
7. S. Hirata and M. Head-Gordon, Time-dependent density functional theory within the Tamm–Dancoff approximation, *Chem. Phys. Lett*, 1999, **314**, 291-299.
8. R. Dennington, T. A. Keith and J. M. Millam, *Semichem Inc.: Shawnee Mission, KS, USA*, 2016.
9. A.-R. Allouche, Gabedit—A graphical user interface for computational chemistry softwares, *J. Comput. Chem*, 2011, **32**, 174-182.
10. W. Humphrey, A. Dalke and K. Schulten, VMD: Visual molecular dynamics, *J. Mol. Graph.*, 1996, **14**, 33-38.
11. J. Edward, F. Ercal, F. G. Walters and H. J. Pottinger, AN EFFICIENT LIBRARY FOR PARALLEL RAY TRACING AND ANIMATION, University of Missouri-Rolla, 1998.
12. V. V. Pavlishchuk and A. W. Addison, Conversion constants for redox potentials measured versus different reference electrodes in acetonitrile solutions at 25°C, *Inorg. Chim. Acta*, 2000, **298**, 97-102.
13. *CrystalClear-SM Expert v2.1*. Rigaku Americas, The Woodlands, Texas, USA, and Rigaku Corporation, Tokyo, Japan,, 2015.
14. *CrysAlisPro v1.171.42.49. Rigaku Oxford Diffraction, Rigaku Corporation, Oxford, U.K., 2022.*
15. M. C. Burla, R. Caliandro, M. Camalli, B. Carrozzini, G. L. Cascarano, C. Giacovazzo, M. Mallamo, A. Mazzone, G. Polidori and R. Spagna, SIR2011: a new package for crystal structure determination and refinement, *J. Appl. Crystallogr.*, 2012, **45**, 357-361.
16. G. Sheldrick, Crystal structure refinement with SHELXL, *Acta Crystallographica Section C*, 2015, **71**, 3-8.

17. O. V. Dolomanov, L. J. Bourhis, R. J. Gildea, J. A. K. Howard and H. Puschmann, OLEX2: a complete structure solution, refinement and analysis program, *J. Appl. Crystallogr.*, 2009, **42**, 339-341.
18. N. G. Connelly and W. E. Geiger, Chemical Redox Agents for Organometallic Chemistry, *Chem. Rev.*, 1996, **96**, 877-910.
19. C. M. Cardona, W. Li, A. E. Kaifer, D. Stockdale and G. C. Bazan, Electrochemical Considerations for Determining Absolute Frontier Orbital Energy Levels of Conjugated Polymers for Solar Cell Applications, *Adv. Mater.*, 2011, **23**, 2367-2371.
20. B. Wang, H. Yang, Y. Zhang, G. Xie, H. Ran, T. Wang, Q. Fu, Y. Ren, N. Sun, G. Zhao, J.-Y. Hu and Q. Wang, Highly efficient electroluminescence from evaporation- and solution-processable orange–red thermally activated delayed fluorescence emitters, *J. Mater. Chem. C.*, 2019, **7**, 12321-12327.
21. B. Zhao, H. Wang, C. Han, P. Ma, Z. Li, P. Chang and H. Xu, Highly Efficient Deep-Red Non-Doped Diodes Based on a T-Shape Thermally Activated Delayed Fluorescence Emitter, *Angew. Chem. Int. Ed.*, 2020, **59**, 19042-19047.
22. C. Zhou, W.-C. Chen, H. Liu, X. Cao, N. Li, Y. Zhang, C.-S. Lee and C. Yang, Isomerization enhanced quantum yield of dibenzo[a,c]phenazine-based thermally activated delayed fluorescence emitters for highly efficient orange OLEDs, *J. Mater. Chem. C.*, 2020, **8**, 9639-9645.
23. C. Li, R. Duan, B. Liang, G. Han, S. Wang, K. Ye, Y. Liu, Y. Yi and Y. Wang, Deep-Red to Near-Infrared Thermally Activated Delayed Fluorescence in Organic Solid Films and Electroluminescent Devices, *Angew. Chem. Int. Ed.*, 2017, **56**, 11525-11529.
24. Y.-L. Zhang, Q. Ran, Q. Wang, Y. Liu, C. Hänisch, S. Reineke, J. Fan and L.-S. Liao, High-Efficiency Red Organic Light-Emitting Diodes with External Quantum Efficiency Close to 30% Based on a Novel Thermally Activated Delayed Fluorescence Emitter, *Adv. Mater.*, 2019, **31**, 1902368.
25. W.-C. Chen, B. Huang, S.-F. Ni, Y. Xiong, A. L. Rogach, Y. Wan, D. Shen, Y. Yuan, J.-X. Chen, M.-F. Lo, C. Cao, Z.-L. Zhu, Y. Wang, P. Wang, L.-S. Liao and C.-S. Lee, Deep-Red/Near-Infrared Electroluminescence from Single-Component Charge-Transfer Complex via Thermally Activated Delayed Fluorescence Channel, *Adv. Funct. Mater.*, 2019, **29**, 1903112.
26. J. Xue, Q. Liang, R. Wang, J. Hou, W. Li, Q. Peng, Z. Shuai and J. Qiao, Highly Efficient Thermally Activated Delayed Fluorescence via J-Aggregates with Strong Intermolecular Charge Transfer, *Adv. Mater.*, 2019, **31**, 1808242.
27. F. Ni, Z. Wu, Z. Zhu, T. Chen, K. Wu, C. Zhong, K. An, D. Wei, D. Ma and C. Yang, Teaching an old acceptor new tricks: rationally employing 2,1,3-benzothiadiazole as input to design a highly efficient red thermally activated delayed fluorescence emitter, *J. Mater. Chem. C.*, 2017, **5**, 1363-1368.
28. Z. Li, D. Yang, C. Han, B. Zhao, H. Wang, Y. Man, P. Ma, P. Chang, D. Ma and H. Xu, Optimizing Charge Transfer and Out-Coupling of A Quasi-Planar Deep-Red TADF Emitter: towards Rec.2020 Gamut and External Quantum Efficiency beyond 30 %, *Angew. Chem. Int. Ed.*, 2021, **60**, 14846-14851.
29. T. Yang, B. Liang, Z. Cheng, C. Li, G. Lu and Y. Wang, Construction of Efficient Deep-Red/Near-Infrared Emitter Based on a Large π-Conjugated Acceptor and Delayed Fluorescence OLEDs with External Quantum Efficiency of over 20%, *J. Phys. Chem. C.*, 2019, **123**, 18585-18592.
30. S. Wang, X. Yan, Z. Cheng, H. Zhang, Y. Liu and Y. Wang, Highly Efficient Near-Infrared Delayed Fluorescence Organic Light Emitting Diodes Using a Phenanthrene-Based Charge-Transfer Compound, *Angew. Chem. Int. Ed.*, 2015, **54**, 13068-13072.
31. Y. Hu, Y. Yuan, Y.-L. Shi, D. Li, Z.-Q. Jiang and L.-S. Liao, Efficient Near-Infrared Emission by Adjusting the Guest–Host Interactions in Thermally Activated Delayed Fluorescence Organic Light-Emitting Diodes, *Adv. Funct. Mater.*, 2018, **28**, 1802597.

32. R. Furue, K. Matsuo, Y. Ashikari, H. Ooka, N. Amanokura and T. Yasuda, Highly Efficient Red–Orange Delayed Fluorescence Emitters Based on Strong π-Accepting Dibenzophenazine and Dibenzoquinoxaline Cores: toward a Rational Pure-Red OLED Design, *Adv. Opt. Mater.*, 2018, **6**, 1701147.
